# The pxn-lgbp-ap-1 pathway restricts virus proliferation by inducing the expression of Cru1 in crayfish

**DOI:** 10.1038/s42003-025-09133-1

**Published:** 2025-12-03

**Authors:** Xiao-Tong Cao, Gang Lu, Lin-Sheng Song, Jie-Jie Sun, Jiang-Feng Lan

**Affiliations:** 1 College of Veterinary Medicine, Shandong Provincial Key Laboratory of Zoonoses, Shandong Agricultural University, Taian, China; 2 Liaoning Key Laboratory of Marine Animal Immunology, Dalian Ocean University, Dalian, China

**Keywords:** Viral host response, Innate immunity

## Abstract

Viruses replicate intracellularly, and extracellular proteins may play a crucial role in preventing viral infections. Peroxinectin (PXN), a myeloperoxidase homolog, is activated extracellularly and possesses peroxidase and cell adhesion activity, defending against bacterial infection through the prophenoloxidase (proPO) system. However, the mechanism of PXN in antiviral immunity requires further study. In this study, PXN was found to be secreted into the hemolymph of crayfish (*Procambarus clarkii*) to recognize VP28 of white spot syndrome virus (WSSV), which then interacts with LPS and β-1,3-glucan binding protein (LGBP) to activate activator protein-1 (AP-1). AP-1 in the nucleus induced the transcription of *crustin1* (*Cru1*). Cru1 exerts its antiviral function by binding to VP28 and subsequently inhibiting the assembly and reinfection of WSSV. These results indicate that the PXN-LGBP-AP-1-Cru1 pathway restricts virus proliferation by inducing the expression of Cru1, representing a mechanism distinct from the previously reported antibacterial immunity mediated by PXN and LGBP.

## Abstract (web summary)

The myeloperoxidase homolog, PXN, is secreted into the hemolymph of crayfish and binds to the white spot syndrome virus (WSSV) protein, VP28, extracellularly. PXN-VP28 then interacts with LGBP, which in turn promotes AP-1 nuclear translocation and expression of Cru1, thereby inhibiting WSSV assembly and crayfish reinfection.

## Introduction

The circulatory system of crustaceans is a semiopen circulatory system, and the study of molecules that perform antiviral functions in the hemolymph may be valuable. White spot syndrome virus (WSSV) is an enveloped double-stranded DNA viral pathogen of crustaceans that has caused considerable economic losses in crustacean farming worldwide^1^. Currently, white spot syndrome is mainly prevented by environmental control. An understanding of antiviral molecules and the mechanism of antiviral infection in crustaceans is urgently needed to develop strategies for the prevention and control of viral diseases.

The prophenoloxidase (proPO) system is a key enzyme cascade system in the innate immunity of invertebrates (especially arthropods)^2,3^. After proPO is activated as phenoloxidase (PO), it primarily functions in the hemolymph and on the cell surface of invertebrates^4–9^. Peroxinectin (PXN), a cell adhesion protein that was originally isolated from *Pacifastacus leniusculus* hemocytes, is synthesized and stored in semigranular and granular hemocytes and is activated by prophenoloxidase-activating enzyme (PPAE) extracellularly, which is related to the activation of the proPO system^10,11^. In response to external stimuli, cell degranulation releases inactive PXN. PXN is activated extracellularly and binds to integrins through the RGD/KGD motif to promote cell adhesion, phagocytosis, and encapsulation for pathogen clearance^12^. Activated PXN also exhibits peroxidase activity and can interact with superoxide dismutase to scavenge pathogenic microorganisms^13^. In previous studies, scientists focused primarily on the function of PXN in antibacterial and antifungal immune defenses (such as degranulation, opsonification, cell adhesion, encapsulation, phagocytosis, and peroxidase activity)^13–16^. However, the mechanism by which PXN regulates intracellular signal transduction remains unclear and needs further elucidation.

There are relatively few studies on the antiviral immune function of PXN. Studies have shown that the expression of PXN is significantly upregulated after stimulation by WSSV^17^. This phenomenon indicates that PXN may be involved in the antiviral immune response process. In the present study, three immune-related proteins from *P. clarkii* were used: PXN, LPS, and β-1,3-glucan binding protein (LGBP), and activator protein-1 (AP-1). The aim was to reveal the mechanism by which PXN mediates immunorecognition of WSSV, and thus to clarify the antiviral mechanism of the PXN-LGBP-AP-1 pathway. PXN recognizes the viral envelope protein VP28 extracellularly and interacts with LGBP, thereby promoting the nuclear translocation of AP-1. AP-1 mediated the expression of Cru1 to inhibit WSSV infection. This study revealed that the PXN-LGBP-AP-1 pathway mediated antiviral immune effects after WSSV infection, which could be helpful for crayfish disease prevention and control as well as the development of antiviral drugs.

## Results

### The antiviral functions of PXN after WSSV challenge

**Fig. 1:**
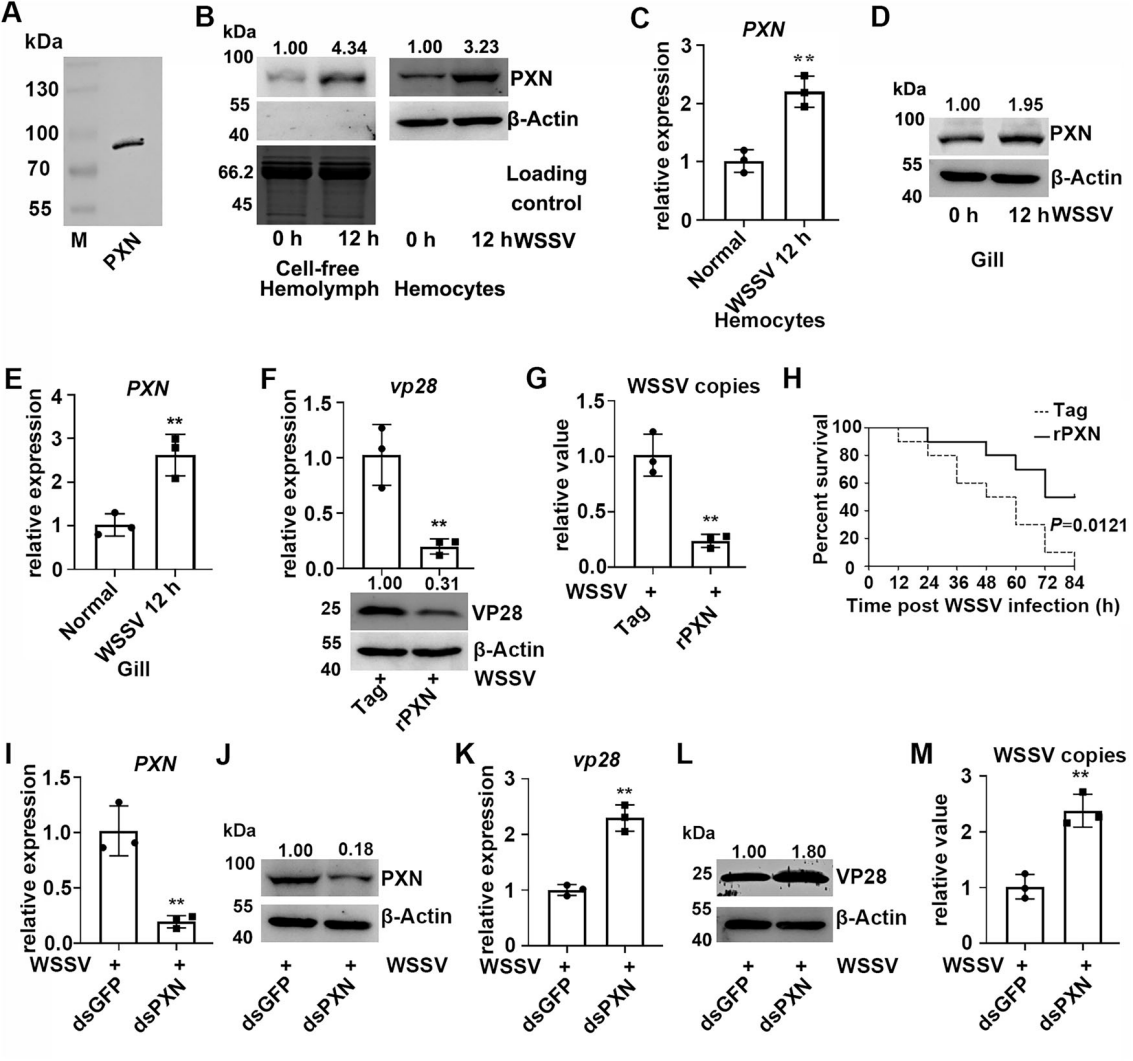
The antiviral functions of PXN in crayfish. **A** PXN in hemocytes was detected by Western blotting using anti-PXN serum as the primary antibody. **B** Protein expression levels of PXN in cell-free hemolymph and hemocytes after WSSV infection. The total protein loading of the cell-free hemolymph was analyzed *via* SDS-PAGE. Gel staining with Coomassie Brilliant Blue was used as the loading control to verify protein loading amounts for cell-free hemolymph samples, while β-Actin served as the internal control for hemocyte samples. **C** The mRNA expression level of PXN in hemocytes after WSSV infection. 18S RNA was used as the internal reference. **D**, **E** The mRNA and protein expression levels of PXN in gills after WSSV infection. β-Actin and 18S RNA served as internal control. **F** The mRNA and protein expression levels of VP28 in rPXN-treated crayfish after WSSV infection. β-Actin and 18S RNA served as internal control. **G** The relative number of WSSV copies in rPXN-treated crayfish after WSSV infection. 18S RNA served as an internal control. **H** Survival rate of crayfish (n = 30) in the rPXN-treated crayfish after WSSV infection. **I**, **J** The mRNA and protein expression levels of PXN in PXN-RNAi crayfish after WSSV infection. β-Actin and 18S RNA served as internal control. **K**, **L** The mRNA and protein expression levels of VP28 in PXN-RNAi crayfish after WSSV infection. β-Actin and 18S RNA served as internal control. **M** The relative number of WSSV copies in PXN-RNAi crayfish after WSSV infection. 18S RNA served as an internal control. For the detection of different proteins (e.g., target protein and internal reference protein), the same protein sample was loaded onto separate gels. After electrophoresis and transfer, different independent blots were obtained. Data are shown as the mean ± SD, (n = 3, each n represents a pooled sample of 5 crayfish). The asterisk (*) represents a significant difference, **P < 0.01 (Student’s *t*-test).

The specificity of anti-PXN, which was prepared in our laboratory (Fig. 1A), was confirmed in hemocytes. To study the antiviral function of PXN, its expression levels were analyzed in cell-free hemolymph, hemocytes, and gills (which are immersed in hemolymph and constitute an important part of the immune system) after WSSV infection. PXN expression increased in cell-free hemolymph, hemocytes, and gills at 12 h after WSSV infection (Fig. 1B–E). To reveal the role of PXN in WSSV infection, rPXN was produced and administered to crayfish hemocoels to simulate an overexpression-like effect. In the rPXN-treated crayfish, both the mRNA and protein expression levels of VP28 were inhibited after WSSV infection (Fig. 1F). The number of WSSV copies was also significantly reduced (Fig. 1G). The survival rate of the rPXN-treated crayfish was significantly greater than that of the Tag-treated crayfish after WSSV infection (Fig. 1H). To verify these results, RNAi was performed to interfere with *PXN* expression. As shown in Fig. 1I, J, the mRNA and protein expression levels of PXN were successfully altered by dsRNA application. In PXN-RNAi crayfish, the mRNA and protein expression levels of VP28 increased significantly after WSSV infection (Fig. 1K, L), and the number of WSSV copies also increased significantly (Fig. 1M). The number corresponding to the top of each band in the Western blot was its relative gray value, which was calculated as described in the “Materials and methods” section.

### Interaction between PXN and VP28 after WSSV infection

**Fig. 2:**
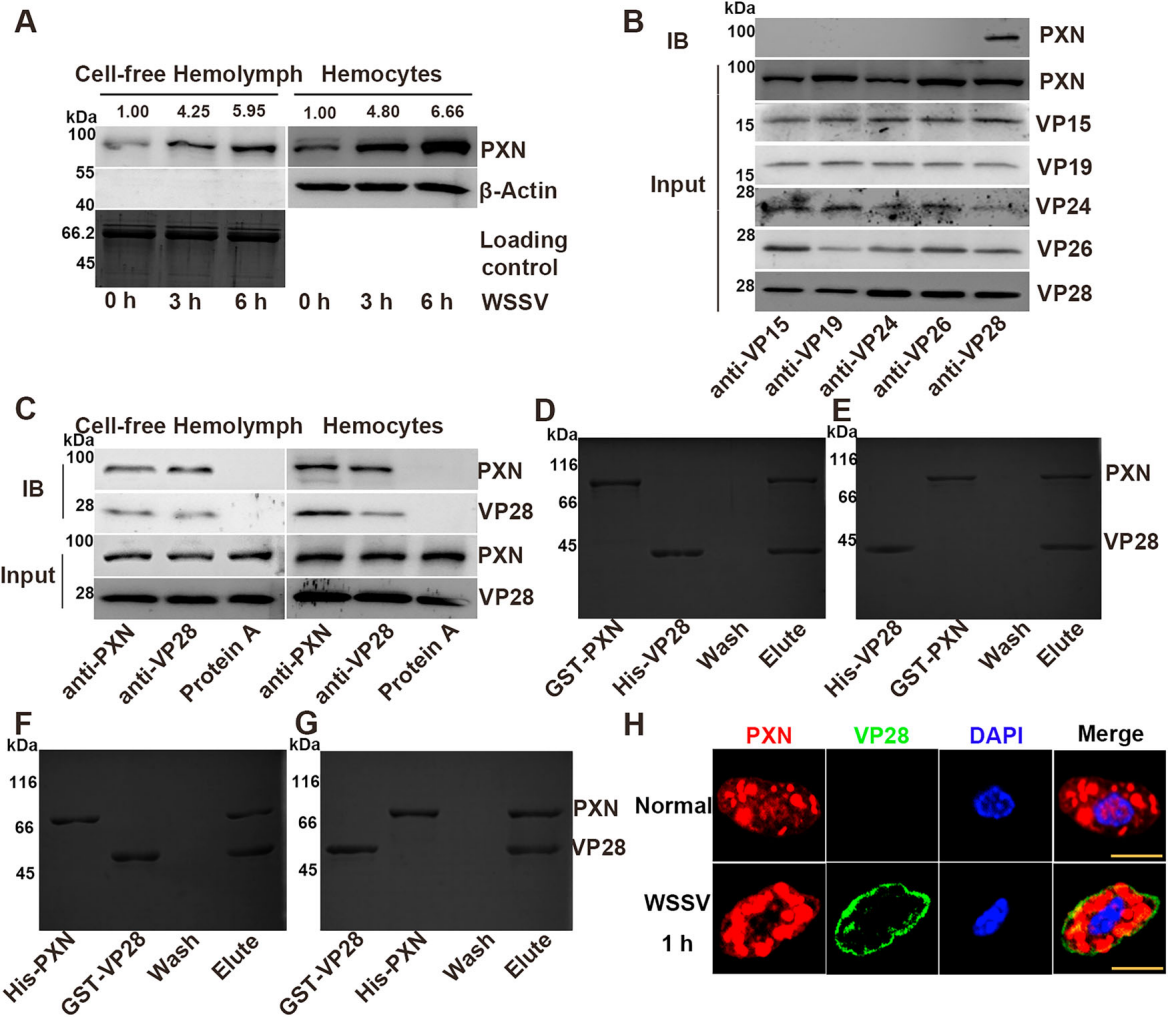
Interaction between PXN and the envelope protein VP28 of WSSV. **A** Protein expression levels of PXN in cell-free hemolymph and hemocytes after WSSV infection. The total protein loading of the cell-free hemolymph was analyzed via SDS-PAGE. Gel staining with Coomassie Brilliant Blue was used as the loading control to verify protein loading amounts for cell-free hemolymph samples, while β-Actin served as the internal control for hemocyte samples. **B** Interaction between WSSV major proteins (VP15, VP19, VP24, VP26, and VP28) and PXN. **C** Interaction between VP28 and PXN in cell-free hemolymph and hemocytes. **D**–**G** Interaction between rPXN and rVP28. **H** Colocalization of PXN and VP28 in hemocytes after WSSV infection. Scale bar = 5 μm. For the detection of different proteins (e.g., target protein and internal reference protein), the same protein sample was loaded onto separate gels. After electrophoresis and transfer, different independent blots were obtained.

Inactive PXN is produced and stored in hemocytes, where it is activated extracellularly. Following WSSV infection, the expression levels of PXN were detected at 0, 3, and 6 h in cell-free hemolymph and hemocytes. The band intensities of the PXN protein increased after WSSV infection in cell-free hemolymph and hemocytes (Fig. 2A). Myeloperoxidase promotes cell adhesion, and opsonin functions to mediate cell-matrix and protein-protein interactions^11,18^. According to previous reports, VP15 (a nucleocapsid protein), VP26 (a tegument protein), VP19, VP24, and VP28 (envelope proteins) are the major structural proteins of WSSV^19,20^. Therefore, to investigate whether PXN recognizes WSSV, the interactions between PXN and WSSV proteins (VP15, VP19, VP24, VP26, and VP28) were analyzed *via* co-IP. A band corresponding to the PXN was detected in the co-IP product of anti-VP28 antibody, indicating an interaction between PXN and VP28. In contrast, no PXN band was observed in co-IP products of antibodies against VP15, VP19, VP24, or VP26 (Fig. 2B). An interaction between PXN and VP28 was detected in the cell-free hemolymph and hemocytes of WSSV-infected crayfish *via* a co-IP assay (Fig. 2C). Bands corresponding to rHis-VP28, rGST-PXN, rGST-VP28, and rHis-PXN were observed after being pulled down by rGST-PXN, rHis-VP28, rHis-PXN, and rGST-VP28, respectively (Fig. 2D–G). Anti-PXN conjugated to Alexa Fluor 647 was observed as red fluorescence, and anti-VP28 conjugated to FITC was observed as green fluorescence. The positive red signals of PXN colocalized with the green signals of VP28 at the cell membrane (Fig. 2H).

### Interaction between PXN and LGBP

**Fig. 3:**
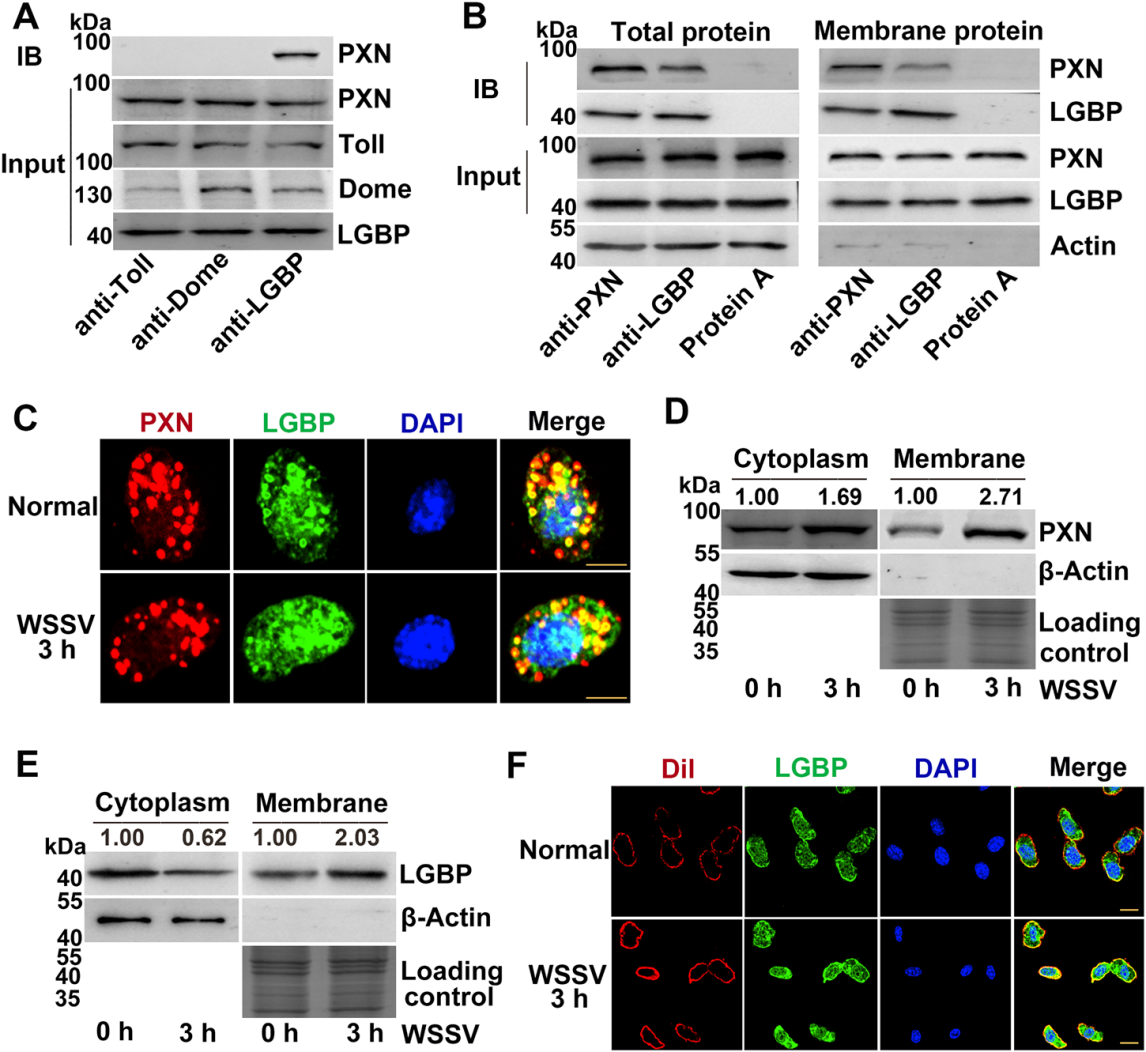
Interaction between LGBP and PXN. **A** Interactions between Toll, Dome, LGBP, and PXN. **B** Interaction between PXN and LGBP. **C** Colocalization of PXN and LGBP in hemocytes. **D** Subcellular localization of PXN in the cytoplasm and membrane after WSSV infection. Gel staining with Coomassie Brilliant Blue was used as the loading control to verify protein loading amounts for membrane samples, while β-Actin served as the internal control for cytoplasm samples. **E** Subcellular localization of LGBP in the cytoplasm and membrane after WSSV infection. Gel staining with Coomassie Brilliant Blue was used as the loading control to verify protein loading amounts for membrane samples, while β-Actin served as the internal control for cytoplasm samples. **F** Colocalization of LGBP and the membrane in hemocytes. Scale bar = 5 μm. The total protein loading of the cell membrane was analyzed via SDS-PAGE. The gel was stained with Coomassie Brilliant Blue as a loading control. For the detection of different proteins (e.g., target protein and internal reference protein), the same protein sample was loaded onto separate gels. After electrophoresis and transfer, different independent blots were obtained.

Pattern recognition receptors (PRRs) constitute the initial step in the defense against pathogen infections. To investigate the immune mechanism regulated by PXN, candidate PRRs (Toll and the key receptor of the proPO-activating cascade (LGBP)) and the classical membrane receptor Domeless of crayfish were screened to analyze the binding of PXN. For hemocyte lysates from crayfish, co-IP assays were performed using specific antibodies against target proteins. A band corresponding to PXN was detected in the co-IP product of the anti-LGBP antibody, indicating a specific interaction between PXN and LGBP. In contrast, no PXN band was observed in co-IP products of anti-Toll or anti-Dome antibodies (Fig. 3A). The interaction between PXN and LGBP was further verified *via* co-IP assay using total protein extracts and membrane protein extracts from crayfish hemocytes. Total protein extracts were used to confirm the presence of the PXN-LGBP interaction, while cell membrane protein extracts were specifically employed to determine whether this interaction occurs at the membrane. The results indicated that PXN and LGBP interact on the cell membrane (Fig. 3B). To verify these results, immunocytochemistry was performed to detect the colocalization of PXN and LGBP using a confocal fluorescence microscope. Anti-LGBP conjugated to FITC was observed as green fluorescence. Anti-PXN conjugated to Alexa Fluor 647 was observed as red fluorescence. The results indicated that PXN and LGBP colocalized in both the cell membrane and the cytoplasm (Fig. 3C). We have demonstrated that PXN and LGBP interact on the cell membrane. To investigate changes in PXN and LGBP on the cell membrane during the early stages of WSSV infection, we conducted the following experiments. Cytoplasmic proteins and membrane proteins were extracted from hemocytes 3 h after WSSV infection, and the expression levels of PXN and LGBP were detected *via* Western blotting. The band intensities of PXN in the cytoplasm and membrane of hemocytes increased after WSSV infection (Fig. 3D). The band intensity of LGBP in the hemocyte membrane increased after WSSV infection (Fig. 3E). Immunocytochemistry was performed in hemocytes to further confirm the relationship between LGBP and the cell membrane. Anti-LGBP conjugated to FITC was observed as green fluorescence, and the membrane stained with Dil was observed as red fluorescence. The results revealed that LGBP colocalized with the cell membrane (Fig. 3F).

### The antiviral functions of LGBP after WSSV infection

**Fig. 4:**
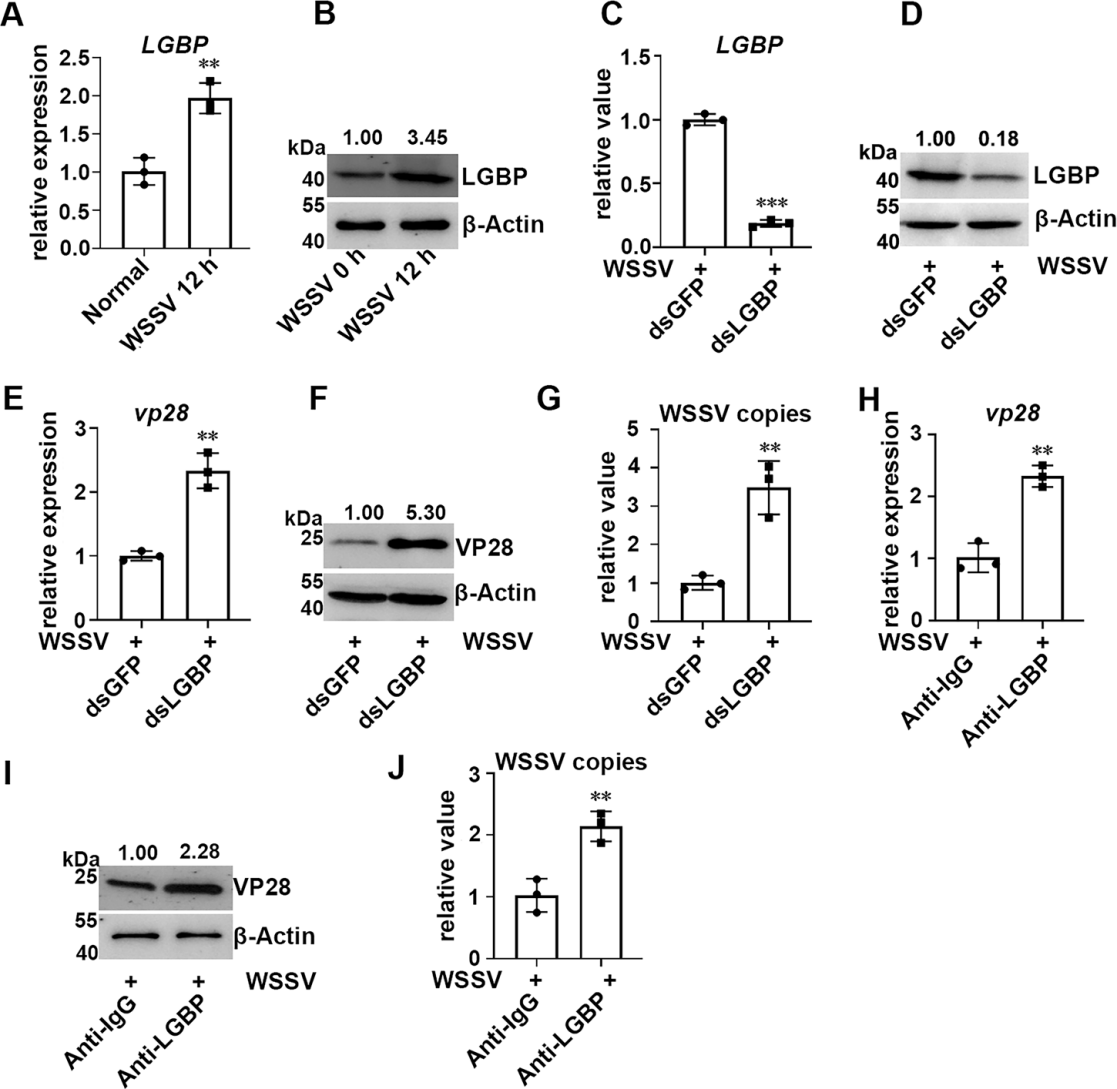
The antiviral functions of LGBP. **A**, **B** mRNA and protein expression levels of LGBP in hemocytes after WSSV infection. β-Actin and 18S RNA served as internal control. **C**, **D** mRNA and protein expression levels of LGBP in LGBP-RNAi-treated crayfish. β-Actin and 18S RNA served as internal control. **E**, **F** The mRNA and protein expression levels of VP28 in LGBP-RNAi crayfish after WSSV infection. β-Actin and 18S RNA served as internal control. **G** The relative value of WSSV copies in LGBP-RNAi crayfish after WSSV infection. 18S RNA served as an internal control. **H**, **I** The mRNA and protein expression levels of VP28 in anti-LGBP-treated crayfish after WSSV infection. β-Actin and 18S RNA served as internal control. **J** The relative number of WSSV copies in anti-LGBP-treated crayfish after WSSV infection. 18S RNA served as an internal control. For the detection of different proteins (e.g., target protein and internal reference protein), the same protein sample was loaded onto separate gels. After electrophoresis and transfer, different independent blots were obtained. Data are shown as the mean ± SD, (n = 3, each n represents a pooled sample of 5 crayfish). The asterisk (*) represents a significant difference, **P < 0.01, ***P < 0.001 (Student’s *t*-test).

WSSV infection induces the expression of LGBP^21,22^. Whether LGBP participates in antiviral reactions has not been systematically reported. The mRNA transcription level of *LGBP* increased significantly (1.97-fold of that at 0 h, *P* < 0.01) in hemocytes at 12 h after WSSV infection (Fig. 4A). The protein level of LGBP also increased (3.45-fold of that at 0 h) in hemocytes at 12 h after WSSV infection (Fig. 4B). An RNAi assay was performed to inhibit the expression of *LGBP*. The mRNA transcription level of *LGBP* decreased significantly (0.19-fold of that in the GFP-RNAi crayfish, *P* < 0.001) in the hemocytes of the LGBP-RNAi crayfish (Fig. 4C). The protein level of LGBP was also reduced (0.18-fold of that in GFP-RNAi crayfish) in hemocytes (Fig. 4D). In LGBP-RNAi crayfish, the mRNA expression level of *vp28* was increased in hemocytes after WSSV infection and was 2.33-fold (*P* < 0.01) of that in GFP-RNAi crayfish (Fig. 4E). The protein level of VP28 was increased (5.30-fold of that in the GFP-RNAi crayfish) (Fig. 4F). The number of WSSV copies also increased significantly, which was 3.48-fold (*P* < 0.01) of that in the GFP-RNAi crayfish (Fig. 4G). In anti-LGBP-treated crayfish, the mRNA transcription level of *vp28* increased significantly after WSSV infection and was 2.33-fold (*P* < 0.01) of that in anti-IgG-treated crayfish (Fig. 4H). The protein level of VP28 was also increased (2.28-fold of that in anti-IgG-treated crayfish) (Fig. 4I). The number of WSSV copies increased significantly in anti-LGBP-treated crayfish, which was 2.14-fold (*P* < 0.01) of that in anti-IgG-treated crayfish (Fig. 4J). In summary, LGBP inhibits WSSV infection.

### Effects of PXN and LGBP on AP-1 nuclear translocation after WSSV infection

**Fig. 5:**
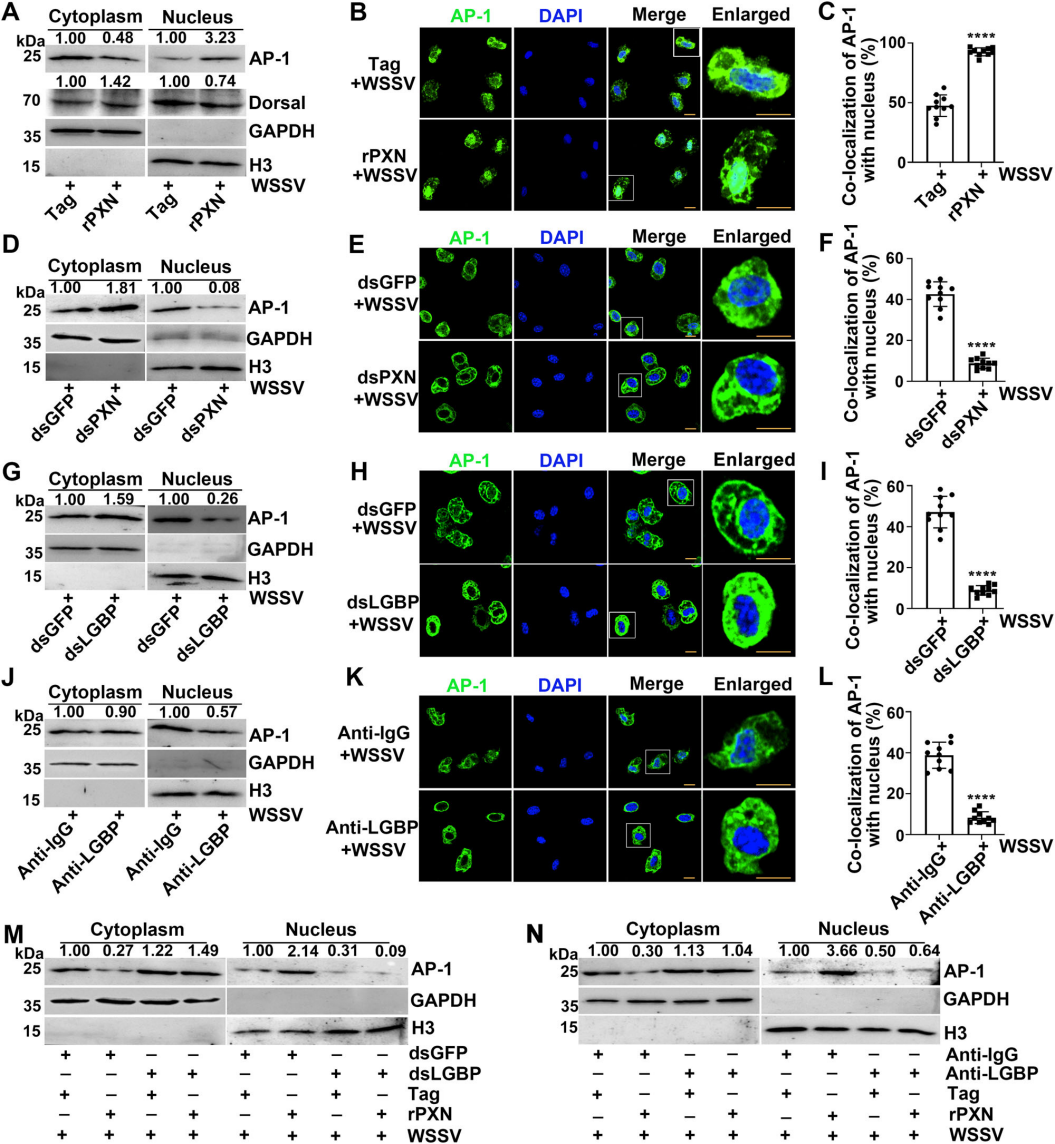
Regulatory effects of PXN and LGBP on the nuclear translocation of AP-1 after WSSV infection. **A** Nuclear translocation of AP-1 and Dorsal in rPXN-treated crayfish after WSSV infection. **B** The nuclear translocation of AP-1 was detected by immunocytochemistry in rPXN-treated crayfish after WSSV infection. **C** Statistical analysis of the colocalization of AP-1 with the nucleus in (**B**). **D**, **E** Nuclear translocation of AP-1 in PXN-RNAi crayfish after WSSV infection. **F** Statistical analysis of the colocalization of AP-1 with the nucleus in (**E**). **G**, **H** Nuclear translocation of AP-1 in LGBP-RNAi crayfish after WSSV infection. **I** Statistical analysis of the colocalization of AP-1 with the nucleus in (**H**). **J**, **K** Nuclear translocation of AP-1 in anti-LGBP-treated crayfish after WSSV infection. **L** Statistical analysis of the colocalization of AP-1 with the nucleus in (**K**). **M**, **N** The subcellular distribution of AP-1 was analyzed in crayfish subjected to LGBP-RNAi or anti-LGBP treatment, followed by rPXN treatment and subsequent WSSV infection. H3 served as the internal control for nucleus samples, while GAPDH served as the internal control for cytoplasm samples. For the detection of different proteins (e.g., target protein and internal reference protein), the same protein sample was loaded onto separate gels. After electrophoresis and transfer, different independent blots were obtained. The percentage of AP-1 colocalized with the nucleus was analyzed *via* ImageJ software. Scale bar = 5 μm. Data are shown as the mean ± SD (n = 10). ****P < 0.0001 (Student’s *t*-test).

The Toll, IMD, and Jak/Stat signaling pathways play crucial roles in innate immunity in both vertebrates and invertebrates by promoting the entry of transcription factors into the nucleus to regulate the transcription of effector molecules^23–25^. AP-1^26^ and NF-κB^27,28^ are the primary transcription factors involved in these three signaling pathways. A PXN injection assay was performed to confirm whether PXN could regulate the nuclear translocation of AP-1 and Dorsal. In the rPXN-treated crayfish, the band intensity of AP-1 protein in the hemocyte nucleus increased (3.23-fold of that in the Tag-treated crayfish) after WSSV infection, whereas no change in the Dorsal band intensity was detected (Fig. 5A). Anti-AP-1 conjugated to Alexa Fluor 488 was observed as green fluorescence, and the nuclei stained with DAPI were observed as blue fluorescence. The colocalization of AP-1 with the hemocyte nucleus was increased in the rPXN-treated crayfish after WSSV infection and was 1.95-fold of that in the Tag-treated crayfish (Fig. 5B, C). In PXN-RNAi crayfish, the band intensity of AP-1 protein was reduced (0.08-fold of that in GFP-RNAi crayfish) in the hemocyte nucleus after WSSV infection (Fig. 5D). The colocalization of AP-1 with the hemocyte nucleus was reduced in PXN-RNAi crayfish after WSSV infection and was 0.21-fold of that in GFP-RNAi-treated crayfish (Fig. 5E, F). In LGBP-RNAi- or anti-LGBP-treated crayfish, the band intensity of AP-1 protein was reduced in the hemocyte nucleus after WSSV infection (Fig. 5G, J). The colocalization values of AP-1 with the hemocyte nucleus were reduced in the LGBP-RNAi- and anti-LGBP-treated crayfish after WSSV infection (Fig. 5H, I, K, L). When rPXN was injected after interference with or blockade of LGBP, the nuclear AP-1 band intensities were lower in hemocytes than in the control group (Fig. 5M, N). These results indicate that PXN synergizes with LGBP to promote the nuclear translocation of AP-1.

### The expression and antiviral functions of Cru1 after WSSV challenge

**Fig. 6:**
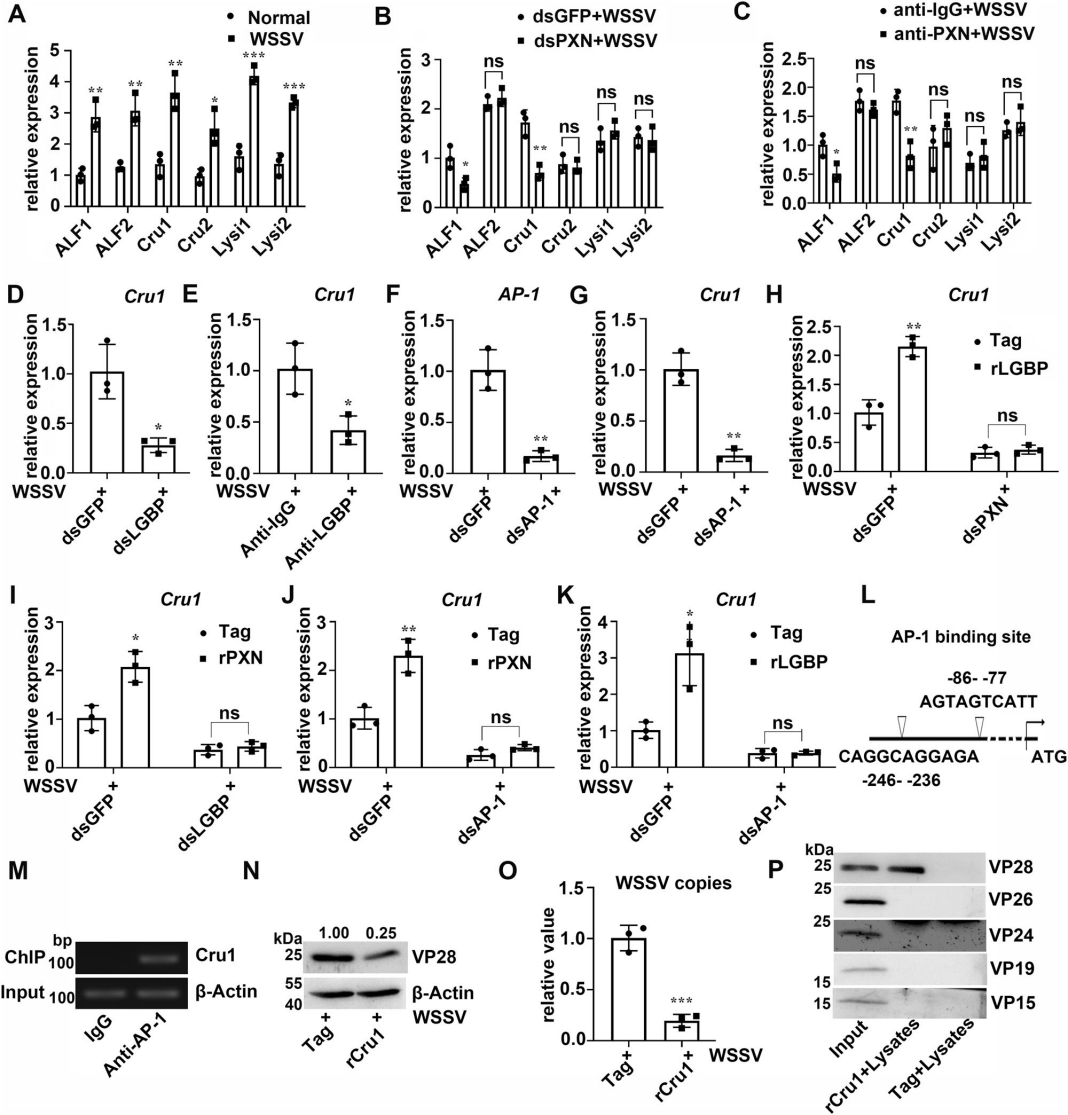
Regulatory effects of PXN and LGBP on Cru1 expression and the antiviral functions of Cru1. **A** mRNA expression levels of AMPs after WSSV infection. 18S RNA served as an internal control. **B** mRNA expression levels of AMPs in PXN-RNAi crayfish after WSSV infection. 18S RNA served as an internal control. **C** mRNA transcription levels of AMPs in anti-PXN-blocked crayfish after WSSV infection. 18S RNA served as an internal control. **D** mRNA transcription level of Cru1 in LGBP-RNAi crayfish after WSSV infection. 18S RNA served as an internal control. **E** mRNA transcription level of Cru1 in anti-LGBP-blocked crayfish after WSSV infection. 18S RNA served as an internal control. **F** The mRNA expression level of AP-1 in AP-1-RNAi crayfish after WSSV infection. 18S RNA served as an internal control. **G** mRNA expression level of Cru1 in AP-1-RNAi crayfish after WSSV infection. 18S RNA served as an internal control. **H** Influence of dsPXN application on rLGBP-enhanced Cru1 expression. Injection of rLGBP and WSSV virions was performed 48 h after dsPXN application. The transcription level of Cru1 was detected 6 h later. 18S RNA served as an internal control. **I** Influence of dsLGBP application on rPXN-enhanced Cru1 expression. Injection of the rPXN and WSSV virions was performed 48 h after dsLGBP application. The transcription level of Cru1 was detected 6 h later. 18S RNA served as an internal control. **J** Influence of dsAP-1 application on rPXN-enhanced Cru1 expression. 18S RNA served as an internal control. **K** Influence of dsAP-1 application on rLGBP-enhanced Cru1 expression. 18S RNA served as an internal control. **L** Analysis of the Cru1 promoter. **M** A ChIP assay was performed to assess the binding of AP-1 to the Cru1 promoter. β-Actin served as an internal control. **N** The protein expression level of VP28 in rCru1-treated crayfish after WSSV infection. β-Actin served as an internal control. **O** Relative number of WSSV copies in rCru1-treated crayfish after WSSV infection. 18S RNA served as an internal control. **P** The interaction between rCru1 and native WSSV (VP15, VP19, VP24, VP26 and VP28) was detected. For the detection of different proteins (e.g., target protein and internal reference protein), the same protein sample was loaded onto separate gels. After electrophoresis and transfer, different independent blots were obtained. Data are shown as the mean ± SD, (n = 3, each n represents a pooled sample of 5 crayfish). *P < 0.05, **P < 0.01, ***P < 0.001, “ns” for no significance *P* > 0.05, (Student’s t-test).

The antilipopolysaccharide factors (ALF1 and ALF2)^29^, crustin (Cru1 and Cru2), and lysozyme (Lysi1 and Lysi2) of crayfish were further screened to confirm the antiviral role of PXN and LGBP. The mRNA expression levels of *ALF1*, *ALF2*, *Cru1*, *Cru2*, *Lysi1*, and *Lysi2* increased significantly after WSSV infection (Fig. 6A). When the expression of *PXN* was interfered with RNAi, the mRNA expression levels of *ALF1* and *Cru1* decreased significantly after WSSV infection and were 0.48-fold and 0.40-fold of that in the GFP-RNAi crayfish, respectively. However, no change was observed in the other four AMPs (Fig. 6B). In anti-PXN-treated crayfish, the *ALF1* and *Cru1* mRNA expression levels decreased significantly after WSSV infection and were 0.51-fold and 0.46-fold of that in anti-IgG-treated crayfish, respectively (Fig. 6C). In our previous studies, ALF1 was shown to be involved in the anti-WSSV immunity of crayfish^30^. To investigate whether Cru1 also exerts anti-WSSV effects, we conducted the following experiments. In LGBP-RNAi- or anti-LGBP-treated crayfish, the mRNA expression level of *Cru1* decreased significantly after WSSV infection and was 0.28-fold of that in GFP-RNAi crayfish and 0.42-fold of that in anti-IgG-treated crayfish (Fig. 6D, E). After *AP-1* was successfully disrupted by dsRNA application (Fig. 6F), the crayfish were infected with WSSV, and the mRNA transcription level of *Cru1* decreased significantly compared with that in the control group (Fig. 6G).

To further confirm that PXN and LGBP regulate the expression of Cru1 through AP-1, the dsRNAs of *PXN*, *LGBP*, or *AP-1* were used to treat crayfish, and then, rLGBP or rPXN was added to detect *Cru1* transcription. The results revealed that after interference with *PXN* or *LGBP*, the expression of *Cru1* was suppressed when LGBP or PXN was replenished, respectively (Fig. 6H, I). Specifically, LGBP was complemented following *PXN* interference, and PXN was complemented following *LGBP* interference. After interference with *AP-1*, the expression of *Cru1* was also suppressed when LGBP or PXN was replenished (Fig. 6J, K). There were two AP-1 binding sites in the *Cru1* promoter region (Fig. 6L), and their interaction was verified through a ChIP assay (Fig. 6M). To study the effect of Cru1 on WSSV replication in crayfish, its recombinant protein was expressed in prokaryotes. Compared with that in the control group, the band intensity of VP28 in the rCru1-treated crayfish decreased after WSSV infection (Fig. 6N), and the number of WSSV copies also decreased (Fig. 6O). The interaction between rCru1 and native WSSV (VP15, VP19, VP24, VP26 and VP28) was detected. The results suggested that Cru1 interacted with VP28 (Fig. 6P). These results indicate that PXN and LGBP participate in antiviral immunity by promoting the nuclear translocation of AP-1, thereby mediating the expression of ALF1 and Cru1.

## Discussion

PXN has been identified in various organisms, including *Drosophila*, crayfish, shrimp, and crab^15,18,31,32^. To date, PXN has been reported to be involved in regulating multiple immune processes to execute antibacterial functions^14,16,31^. Some reports have shown that the mRNA expression of *PXN* can be induced after WSSV infection in crustaceans^15–17^. In the present study, the PXN-LGBP-AP-1 antiviral signaling pathway was identified, and its antiviral immune effectors were confirmed in crayfish.

PXN is the first cell adhesion molecule cloned from crustacean hemolymph^31^. Hemolymph plays crucial roles, such as tolerance to heavy metals in crustaceans^33^ and defense against pathogenic microorganisms. PXN is produced in hemocytes, synthesized in semigranular and granular hemocytes, stored in secretory granules, and released during degranulation^11^. In our study, the PXN protein was distributed in the gills, hemocytes, and cell-free hemolymph. The mRNA and protein expression levels of PXN increased after WSSV infection. Further studies revealed that PXN inhibited the replication of WSSV and increased the survival rate of crayfish. These results suggest that PXN plays a crucial role in crayfish’s antiviral immunity.

As a cell adhesion protein, PXN can recognize different pathogens. For example, rPXN from crabs can bind to *Bacillus subtilis*, *Staphylococcus aureus*, *Aeromonas hydrophila*, and *Vibrio parahaemolyticus*^34^. In the present study, PXN was found to interact directly with VP28 of WSSV. The colocalization of PXN with WSSV was also observed in hemocytes and cell-free hemolymph. Our study demonstrated that PXN can bind the envelope protein of WSSV in cell-free hemolymph and transport signals into hemocytes.

Cell-surface proteins play a crucial role in this signaling process. PRRs are essential molecules for activating the immune system. In crustaceans, LGBP, a PRR, was first purified from *P. leniusculus*, and the activation of proPO was blocked following treatment with LGBP antiserum^35^. In *L. vannamei*, interference with *LGBP* increased the mortality rate of shrimp^22^. This finding was consistent with our results, which showed that LGBP inhibited WSSV replication in crayfish. In crustaceans, LGBP triggers a serine protease cascade by interacting with LPS and β-1,3-glucans, thereby activating the proPO cascade and contributing to antibacterial immunity^36^. In *P. stylirostris*, WSSV infection induced the expression of LGBP, thereby activating the proPO cascade^21^. In *P. leniusculus*, PXN interacts with LGBP through the cell-surface receptor superoxide dismutase to mediate cell adhesion and phagocytosis^13^. Recent research has shown that during pathogen infection, heat shock protein 70 (*Lv*HSP70) is released into the extracellular space, where it interacts with the membrane-localized protein *Lv*LGBP. The interaction between *Lv*HSP70 and *Lv*LGBP triggers a serine protease cascade, leading to the production of melanin, which defends against pathogen infection^37^. In the present study, during WSSV infection, PXN in crayfish recognized WSSV VP28 extracellularly and subsequently directly interacted with LGBP to exert antiviral immune functions, representing a mechanism by which PXN and LGBP participate in antiviral immunity.

In crustaceans, the Toll, IMD, and Jak/Stat signaling pathways play crucial roles in innate immunity^38–41^. Among these genes, AP-1 and Dorsal are important transcription factors. In the present study, rPXN induced the nuclear translocation of AP-1 but did not affect Dorsal, indicating that PXN might resist WSSV infection by regulating the nuclear translocation of AP-1. Furthermore, when the expression of LGBP was inhibited or blocked by anti-LGBP, the nuclear translocation of AP-1 was inhibited in the rPXN-treated crayfish, indicating that LGBP was necessary for the PXN-induced nuclear translocation of AP-1. AP-1 has been reported to play a crucial role in antiviral infections in crustaceans^26^. AMPs (ALFs, Crus, and LYZs) reportedly act as immune effectors to directly execute antiviral functions^30,40,42^. Hemocyanin modulates the expression levels of AMPs (ALFs, Crus, and PENs) by promoting the phosphorylation of proteins in the MKK4-p38-c-Jun signaling pathway, thereby increasing resistance to pathogen infection^43^. In silkworms, after interference with peroxinectin (*BmPxt*), the Jak/Stat, IMD, and Toll signaling pathways are inhibited, and the expression levels of AMPs decrease ^44,45^. In the present study, the mRNA expression of various AMPs was induced after WSSV infection. ALF1 has been shown to be involved in the anti-WSSV immunity of crayfish^30^. We obtained purified Cru1 protein and verified its function. Cru1 inhibited the expression of VP28 and the number of WSSV copies by binding to the envelope protein WSSV VP28. WSSV completes its assembly in the nucleus^46,47^. Cru1 blocked WSSV virion assembly in the nucleus and inhibited WSSV reinfection outside the cell. The enhancement of *Cru1* transcription by PXN and LGBP disappeared after interference with *AP-1*. These results indicate that PXN and LGBP participate in antiviral immunity by promoting the nuclear translocation of AP-1, thereby mediating the expression of Cru1.

**Fig. 7:**
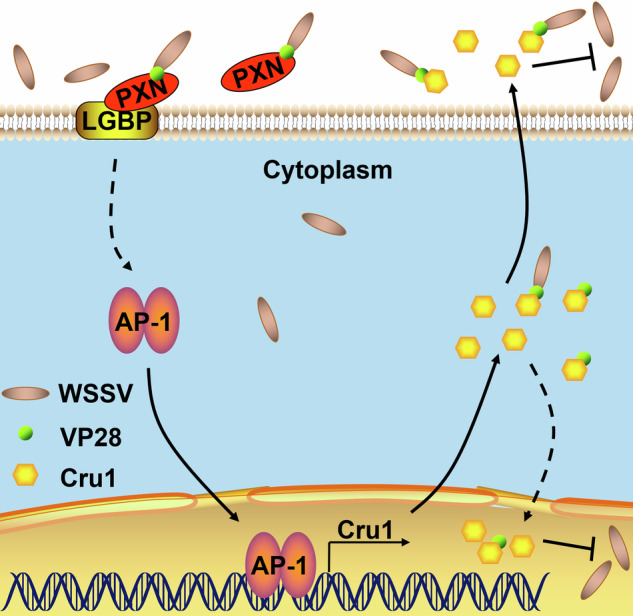
Working model of the PXN-mediated antiviral immune response in crayfish. PXN recognizes WSSV by binding to the envelope protein (VP28). PXN interacts with LGBP, leading to the nuclear translocation of AP-1. Activated AP-1 regulates the transcription of *Cru1*. Cru1 inhibits the assembly and reinfection of WSSV.

In conclusion, PXN can be secreted into cell-free hemolymph as an extracellular ligand after WSSV infection and binds to the envelope protein VP28, which then transfers signals into the intracellular receptor LGBP to induce the nuclear translocation of AP-1. AP-1 in the hemocyte nucleus can bind the promoter region of *Cru1* to induce the transcription of *Cru1*. The produced Cru1 could execute antiviral functions by inhibiting the assembly and reinfection of WSSV (Fig. 7). The PXN-LGBP-AP-1-Cru1 axis represents a potential therapeutic target for enhancing antiviral responses in crustaceans. Furthermore, this axis defines a distinct antiviral mechanism, which differs from the antibacterial immunity that PXN and LGBP mediate.

## Materials and methods

### Animals and sample collection

The crayfish *P. clarkii* (10–15 g each) was obtained from a fish market in Tai’an, Shandong Province, China, and raised in aerated water at 22 °C for at least one week before the subsequent experiment. The crayfish (similar in color, size, and health condition, sex was not considered) were randomly divided into the experimental group and the control group, with each group composed of at least 5 crayfish. Total RNA was extracted from different crayfish tissues using TRIzol reagent (Vazyme, China), and genomic DNA was extracted using a genomic DNA extraction kit (Vazyme, China) according to the manufacturer’s instructions. cDNA was synthesized *via* a cDNA synthesis kit (Vazyme, China) according to the manufacturer’s instructions. Hemocytes and gills were collected and then washed with PBS (137 mM NaCl, 2.7 mM KCl, 2 mM KH_2_PO_4_, and 10 mM Na_2_HPO_4_, pH 7.4) three times to detect the expression of PXN in WSSV-infected crayfish. The 3-6-month-old specific pathogen-free (SPF) New Zealand White rabbits (Oryctolagus cuniculus) were purchased from a market in Tai’an for antibody preparation. The rabbits were housed in standard cages at 23 °C, and were fed rabbit food and provided with water twice daily. The antibody preparation method is described in the section on preparation of the recombinant protein and antibody. The study was approved by the Committee on the Animal Ethics of Shandong Agricultural University. The experiments were carried out in accordance with the approved guidelines (No. SDAUA-2024-065).

### mRNA expression analysis of target genes *via* quantitative real-time PCR (qRT-PCR)

QRT-PCR with primers (Supplementary Table 1) was performed to detect the mRNA transcripts of target genes in the tissues of hemocytes or gills. The 18S rRNA gene fragments amplified with the primers 18S-RTF/R (Supplementary Table 1) were used as the internal reference. The qRT-PCR procedure was as follows: 94 °C for 2 min, followed by 40 cycles of 94 °C for 15 s and 60 °C for 30 s. The relative mRNA expression levels of the target genes were analyzed *via* the 2^−ΔΔCt^ method^48^. The RNA extracted from each experimental group was a mixed sample of at least 5 crayfish.

### Preparation of the recombinant protein and antibody

The sequences of the peroxidase domain in PXN (ADW79421.1) and Cru1 (ACY64751.1) were amplified from hemocytes *via* the primer pairs PXN-EF/R and Cru1-EF/R (Supplementary Table 1), respectively. The DNA fragments were ligated into the pET-30a or pGEX4T-1 vector to construct recombinant plasmids. The plasmids pET-30a-PXN, pGEX4T-1-PXN, and pGEX4T-1-Cru1 were transformed into BL21 *Escherichia coli cells* (Sangon, China). The positive transformants were cultured, and the recombinant protein was induced by adding isopropyl-β-D-thiogalactopyranoside (IPTG) at a final concentration of 0.5 mM. The inclusion bodies of the expressed proteins were washed, dissolved, and dialyzed in 1 L of dialysate (5 mM EDTA, 8 M urea, 0.6 mg/ml L-cysteine, 100 mM Tris-HCl, pH 8.0) and PBS for 12 h, respectively. The antiserum was prepared^49,50^. Purified recombinant PXN protein (rPXN) was used to produce a polyclonal antibody in rabbits. Purified rPXN (200 μg) was mixed with complete Freund’s adjuvant at a 1:1 volume ratio. The emulsified mixture was administered to the rabbit via subcutaneous multiple-point injection. Two to three weeks after the first injection, the rabbit received an emulsified mixture with incomplete Freund’s adjuvant for booster immunization. Serum samples were collected from the rabbits via multiple blood draws from the marginal ear vein following the third booster vaccination. After an observation period, the rabbits were transferred to a specialized facility for further care and management. Blood samples were incubated at 4 °C to separate the serum. Hemocytes were collected from crayfish, and the target protein in the hemocytes was detected via Western blotting to verify the specificity of antibodies.

### Determination of target proteins by Western blotting analysis

The protein expression levels of PXN, LGBP (ACR20474.1), AP-1, and VP28 were analyzed *via* Western blotting. The extraction of membrane, cytoplasmic, and nuclear proteins was accomplished via a Membrane Protein Extraction Kit and a Nuclear Protein Extraction Kit (Beyotime, China), following the manufacturer’s instructions. For analysis of cell membrane proteins, cytoplasmic and cell membrane proteins were extracted from hemocytes at 0 and 3 h post-WSSV infection. As there are no commercial cell membrane or hemolymph reference proteins available for crayfish, we analyzed the total protein loading in cell membranes (as well as cell-free hemolymph) via sodium dodecyl sulfate-polyacrylamide gel electrophoresis (SDS-PAGE). Gel staining with Coomassie Brilliant Blue was used as the loading control to verify protein loading amounts for membrane (as well as cell-free hemolymph) samples, while β-Actin served as the internal control for cytoplasm samples. For the analysis of nuclear proteins, cytoplasmic and nuclear proteins were extracted from hemocytes at 6 h post-WSSV infection (after the first post-injection) to detect the nuclear translocation of AP-1. GAPDH and H3 served as internal reference proteins for cytoplasmic and nuclear proteins, respectively. The prepared protein samples (15 μL) were separated via SDS-PAGE and transferred to nitrocellulose (NC) membranes. The membrane was blocked with 3% nonfat milk (diluted in TBS containing 150 mM NaCl and 10 mM Tris-HCl, pH 8.0) for 1 h and then incubated with the primary antibody (1:250 dilution) at 4 °C overnight. After being washed three times with TBST (0.02% Tween-20 in TBS) and TBS, the membrane was incubated with HRP-conjugated goat anti-rabbit IgG (1:10,000 dilution) (Proteintech, China) for 1 h. After being washed in the same way, the signals were examined via an enhanced chemiluminescence detection assay kit (CWBIO, China). For the detection of different proteins (e.g., target protein and internal reference protein), the same protein sample was loaded onto separate gels. After electrophoresis and transfer, different independent blots were obtained. The quantitative value of the destination strip = (total gray value of each destination band/total gray value of the internal reference band corresponding to each destination band) × 100%. The gray value of the control group was labeled as 1.0.

### RNA interference (RNAi) and antibody blockage assay

The RNAi assay was performed by injecting double-stranded RNA (dsRNA) targeting *PXN*, *LGBP*, and *AP-1*. The cDNA fragments of the *PXN*, *LGBP*, and *AP-1* genes were amplified *via* their corresponding primers (PXN-RNAiF/R, LGBP-RNAiF/R, and AP-1-RNAiF/R) (Supplementary Table 1), which were linked to the T7 promoter. A commercial transcription T7 kit (Thermo, America) was used to synthesize dsRNAs of *PXN*, *LGBP*, and *AP-1* (dsGFP as the control). The crayfish in each group received an individual injection of 30 μg of dsRNA, and total RNA and protein were extracted to evaluate RNAi efficacy at 48 h after the injection. The RNAi-treated crayfish received an injection of WSSV (50 μL, approximately 1 × 10^4^ virions), and the hemocytes were extracted to analyze the nuclear translocation of AP-1 at 6 h after the second injection. The hemocytes were collected to examine the mRNA and protein expression of VP28, as well as the relative number of WSSV copies at 48 h after the second injection. The crayfish received an injection of anti-PXN or anti-LGBP (30 μg) to block the protein activity of PXN or LGBP, respectively. The anti-PXN- or anti-LGBP-treated crayfish received an injection of WSSV (50 μL, approximately 1 × 10^4^ virions), and the hemocytes were collected to analyze the mRNA and protein expression levels of VP28 and the relative value of WSSV copies at 48 h after the second injection.

### Treatment with rPXN and rCru1

The crayfish in each group individually received an injection of 30 μg of rPXN or rCru1, with GST-Tag as the control. 2 h after the injection of the recombinant protein, the crayfish received an injection of WSSV (50 μL, approximately 1 × 10^4^ virions). Total RNA, protein, and genomic DNA were extracted to analyze WSSV replication levels *via* qRT-PCR and Western blotting at 48 h after WSSV injection.

### Crayfish survival assay

Thirty crayfish received an injection of 30 μg of rPXN protein or Tag protein (control group). The crayfish in both groups received an injection of WSSV (50 μL, approximately 1 × 10^4^ virions) at 2 h after the injection of the recombinant protein. The number of live crayfish was recorded every 12 h. The data were analyzed *via* GraphPad Prism software.

### Pulldown assay

A pulldown assay was performed^51^. A GST pulldown assay was used to determine the in vitro interaction between PXN and VP28. The purified rGST-PXN (1 mg/mL, 150 μL) and 10 μL of GST-binding resin (GenScript, China) were incubated at 4 °C for 30 min, washed three times with PBS, and then incubated with the purified rHis-VP28 (1.5 mg/mL, 200 μL) at 4 °C for 2 h. The reaction mixture was washed with PBS three times. The collected samples were analyzed *via* 12% SDS-PAGE. His pulldown was performed *via* the same method described above to further confirm the interaction between rHis-PXN and rGST-VP28. The interaction between rHis-PXN and rGST-VP28 was studied *via* a method similar to that described above.

### Coimmunoprecipitation (co-IP) assay

The co-IP assay was used to screen for WSSV major proteins (VP15, VP19, VP24, VP26, and VP28) interacting with PXN. Protein A beads (Sangon, China) were washed three times with PBS *via* centrifugation at 3000 rpm for 5 min (4 °C). Then, 1 μg of each specific antibody (anti-VP15, anti-VP19, anti-VP24, anti-VP26, and anti-VP28 antibodies) was added to individual tubes and incubated at 4 °C for 1 h. After that, 1 mL of hemolysate (prepared from WSSV-infected crayfish at 48 h post-infection) was added to each tube, followed by incubation at 4 °C for 2 h. The beads were then washed three times with PBS to remove unbound proteins, and the resulting pellet was resuspended in electrophoresis sample buffer and denatured for 5 min. The protein samples were separated by SDS-PAGE and analyzed by Western blotting using an anti-PXN antibody. Using a similar approach described above, we further screened for protein-protein interactions between PXN and Toll, Dome, or LGBP.

A co-IP assay was performed to examine the interaction between PXN and VP28 or LGBP in vivo. Ten microliters of Protein A resin was mixed with 1 mL of PBS in EP tubes. After being washed three times with PBS by centrifugation at 3000 rpm and 4 °C for 5 min, anti-PXN and anti-VP28 (1 μg) were individually added to the tubes and incubated at 4 °C for 1 h. Then, hemocytes and cell-free hemolymph were added to the tubes and incubated at 4 °C for 2 h, respectively. After washing with PBS three times, the resulting pellet (containing bound protein, Ab, and Protein A) was added to the electrophoresis sample buffer and denatured for 5 min. The protein samples were analyzed *via* SDS-PAGE and Western blotting with anti-PXN and anti-VP28 antibodies, respectively. The interaction between PXN and LGBP in vivo was studied *via* a method similar to that described above. The protein samples were finally analyzed *via* Western blotting with anti-PXN or anti-LGBP antibodies.

### Far Western blot assay

Far Western blot assay was performed to detect whether rCru1 could interact with WSSV major proteins (VP15, VP19, VP24, VP26, and VP28). Approximately 200 μg of purified GST-Cru1 was incubated with 20 μL of GST-binding resin at 4 °C for 1 h. The resin was then washed three times with PBS *via* centrifugation at 3000 rpm for 5 min (4 °C), followed by the addition of hemocyte lysate from WSSV-infected crayfish (1 mL) and further incubation at 4 °C for 2 h. Finally, the resin was eluted using elution buffer (10 mM reduced glutathione, 50 mM Tris-HCl, pH 8.0). The eluted samples were then analyzed by Western blotting using antiserum against VP15, VP19, VP24, VP26, and VP28.

### Immunocytochemistry assay

The hemolymph was extracted and mixed with 1 mL of cold anticoagulant. After centrifugation, the hemocytes were incubated with cold anticoagulant containing 4% paraformaldehyde at 4 °C for 10 min. The hemocytes were resuspended in PBS and then dropped onto polylysine-coated glass slides. The slides were incubated with PBS containing 0.2% Triton X-100 for 5 min. After being washed three times with PBS, the slides were blocked with 3% nonfat milk (dissolved in PBS) at 4 °C for 1 h The hemocytes were then incubated with anti-AP-1 (1:100 dilution) as the primary antibody and goat anti-rabbit Alexa Fluor 488 (Abbkine, America) (1:1000 dilution) as the secondary antibody in the dark at 4 °C overnight. After washing with PBS three times, the hemocyte nuclei were stained with 4′,6-diamidino-2-phenylindole (DAPI). After being washed three times with PBS, the slides were observed under a fluorescence microscope^30,52^. The percentage of AP-1 colocalized with the nucleus was analyzed *via* the Wright Cell Imaging Facility ImageJ software.

The colocalization of LGBP with the cell membrane was investigated *via* a method similar to that described above. Before the hemocyte nuclei were stained with DAPI, the hemocyte membrane was stained with Dil (Beyotime, China).

An immunocytochemistry assay was conducted to determine the colocalization of PXN with VP28 or LGBP. Hemocytes were collected from crayfish at 1 h after WSSV infection, and those from untreated crayfish (normal group) were used as controls. The colocalization of PXN and VP28 was examined with Fluor 647 (Frdbio, China)-labeled PXN and fluorescein isothiocyanate (FITC, Sigma, America)-labeled VP28 antiserum. Hemocytes were collected from crayfish at 3 h after WSSV infection, and the colocalization of PXN and LGBP was analyzed with Fluor 647-labeled PXN and FITC-labeled LGBP antiserum^53^.

### Chromatin immunoprecipitation (ChIP) assay

The ChIP assay was performed^54,55^. Hemocytes were collected at 6 h after WSSV infection and fixed with 1% paraformaldehyde at room temperature for 10 min. Glycine (final concentration: 0.2 M) was then added to quench the fixation reaction. After being washed three times with PBS at 4 °C, the SDS lysis buffer (10 mM Tris-HCl (pH 7.5), 150 mM NaCl, 1% SDS, 1 mM EDTA) was added to resuspend the samples. Samples were sonicated on ice and then centrifuged at 12,000 rpm for 15 min at 4 °C to remove impurities. The supernatant was diluted with immunoprecipitation (IP) buffer (Beyotime, China). Subsequently, the sample was incubated with pre-immune serum (negative control, anti-IgG) and anti-AP-1 antibody (experimental group) at 4 °C for 3 h, respectively. Protein A beads were added, and the incubation was continued for an additional 3 h. The beads were washed three times with IP dilution buffer, three times, twice with 250 mM LiCl buffer, twice with Tris-EDTA buffer. The immune precipitate was eluted by elution buffer (25 mM Tris-HCl (pH 7.5), 10 mM EDTA, 0.5% SDS) at 65 °C for 15 min. The samples were then incubated with proteinase K at 42 °C for 2 h, followed by incubation at 65 °C for 5 h. DNA was extracted using phenol-chloroform. The supernatant was mixed with anhydrous ethanol (2.5 volumes) and 3 M sodium acetate (0.1 volume), then incubated at −20 °C for 2 h to precipitate DNA. The DNA pellet was washed with 75% ethanol. Finally, the precipitated DNA was dissolved in nuclease-free water. The primers used for amplifying the Cru1 promoter are listed in Supplementary Table 1.

### Statistics and reproducibility

Statistical analyses were performed using GraphPad Prism 8.0 software. Data are shown as the mean ± SD. The asterisk (*) represents a significant difference, *P < 0.05, **P < 0.01, ***P < 0.001, ****P < 0.0001, or “ns” for no significance, P > 0.05, as analyzed using Student’s *t*-test. The gray value of the band was analyzed using the Wright Cell Imaging Facility ImageJ software. The domain architecture was predicted using SMART. The promoter sequence was analyzed using Promoter Scan online (https://www-bimas.cit.nih.gov/molbio/proscan/). The raw data are presented in “Supplementary Tables and Fig. 1” and “Supplementary Data 1”. At least three independent experiments confirmed the reproducibility of our results.

### Reporting summary

Further information on research design is available in the Nature Portfolio Reporting Summary linked to this article.

## Supplementary information

Supplementary Information

Description of Additional Supplementary Files

Supplementary Data 1

Reporting summary

## Data Availability

All raw data are available in Supplementary Material named “Supplementary Tables and Fig. 1” (including Supplementary Table 1, the uncropped blots, gels, and immunofluorescence images. The uncropped blots, gels, and immunofluorescence images in Supplementary Figs. 1–6 correspond one-to-one with the blots, gels, and immunofluorescence images in Figs. 1–6 of the paper) and Data Sets named “Supplementary Data 1” (numerical source data). All other supporting data are available from the corresponding author upon reasonable request.
