# Supplementary material for: The pxn-lgbp-ap-1 pathway restricts virus proliferation by inducing the expression of Cru1 in crayfish
Source: Commun Biol. 2025 Dec 3;8:1742. doi: 10.1038/s42003-025-09133-1 (PMC12675793; doi:10.1038/s42003-025-09133-1)
Supplement: Supplementary file 1 — Supplementary Information [file 42003_2025_9133_MOESM1_ESM.pdf]

Supplementary Table 1

STable 1. The sequences of the primers used in the study

Primer	Sequence（5′-3′）
qRT-PCR	
PXN-RTF	CAGTTCGCTCGCCTCAA
PXN-RTR	GCAGTGTCGCAGTAGATCC
VP28-RTF	AGCTCCAACACCTCCTCCTTCA
VP28-RTR	TTACTCGGTCTCAGTGCCAGA
18S-RTF	TCTTCTTAGAGGGATTAGCGG
18S-RTR	AAGGGGATTGAACGGGTTA
LGBP-RTF	GGGAGAATGGTGAGGGC
LGBP-RTR	CATCAGCAGAGGAAGAGGC
ALF1-RTF	GAAGCGATGACGAGGAGCAAT
ALF1-RTR	GACGGGTTGGCACAAGAGC
ALF2-RTF	CAAACTGGGCGGGTTATGG
ALF2-RTR	TGACGAAGTCCCTGGTGGC
Cru1-RTF	TATTCCTCGCTGCACAAACA
Cru1-RTR	CACATAGCACCTCCCTCTTCA
Cru2-RTF	CGCACAGCCGAGAGAAACACTATCAAGAT
Cru2-RTR	GGCCTATCCCTCAGAACCCAGCACG
Lysi1-RTF	GTCAACCCACCCTCAATAAC
Lysi1-RTR	CTTGTGAATCAGGGCGTA
Lysi2-RTF	ATCAAAGGCGACGATAAGACG
Lysi2-RTR	TGTGTATGCGAGCCAAGTCAA
AP-1-RTF	CTGAGTCCGTCCGATAAGTG
AP-1-RTR	GTGGCGGTTTTGGTGGGGAAGAAGA
RNAi	
PXN-RNAiF	GCGTAATACGACTCACTATAGGCTAACCATTCCCCTAC
PXN-RNAiR	GCGTAATACGACTCACTATAGGAGATTCCACTTCTGTCA
LGBP-RNAiF	GCGTAATACGACTCACTATAGGAACCGCAGCATCAGTTACAC
LGBP-RNAiR	GCGTAATACGACTCACTATAGGGGTTGTCCAGGGAGTTGTCG
AP-1-RNAiF	GCGTAATACGACTCACTATAGGGGCTGAGCGCCGCTGTACCG
AP-1-RNAiR	GCGTAATACGACTCACTATAGGATATACATACATAATTTATA
GFP-RNAiF	GCGTAATACGACTCACTATAGGTGGTCCCAATTCTCGTGGAAC
GFP-RNAiR	GCGTAATACGACTCACTATAGGCTTGAAGTTGACTTGATGCC
Expression	
PXN-EF	GATGAATTCAGGTGCACCCCCAACAGC
PXN-ER	GACCTCGAGCTACACGCCCGGCCTCTC
Cru1-EF	TACTCAGGATCCATGGTGGTGATGGCGATT
Cru1-ER	TACTCAGAATTCTCAGACCTCAGAGCTACGACAAATG
ChIP	
Cru1-ChIP-F	CCACAGAAGATGCACAAGA
Cru1-ChIP-R	CAACTACAGTACTATACCC

Supplementary Figure 1

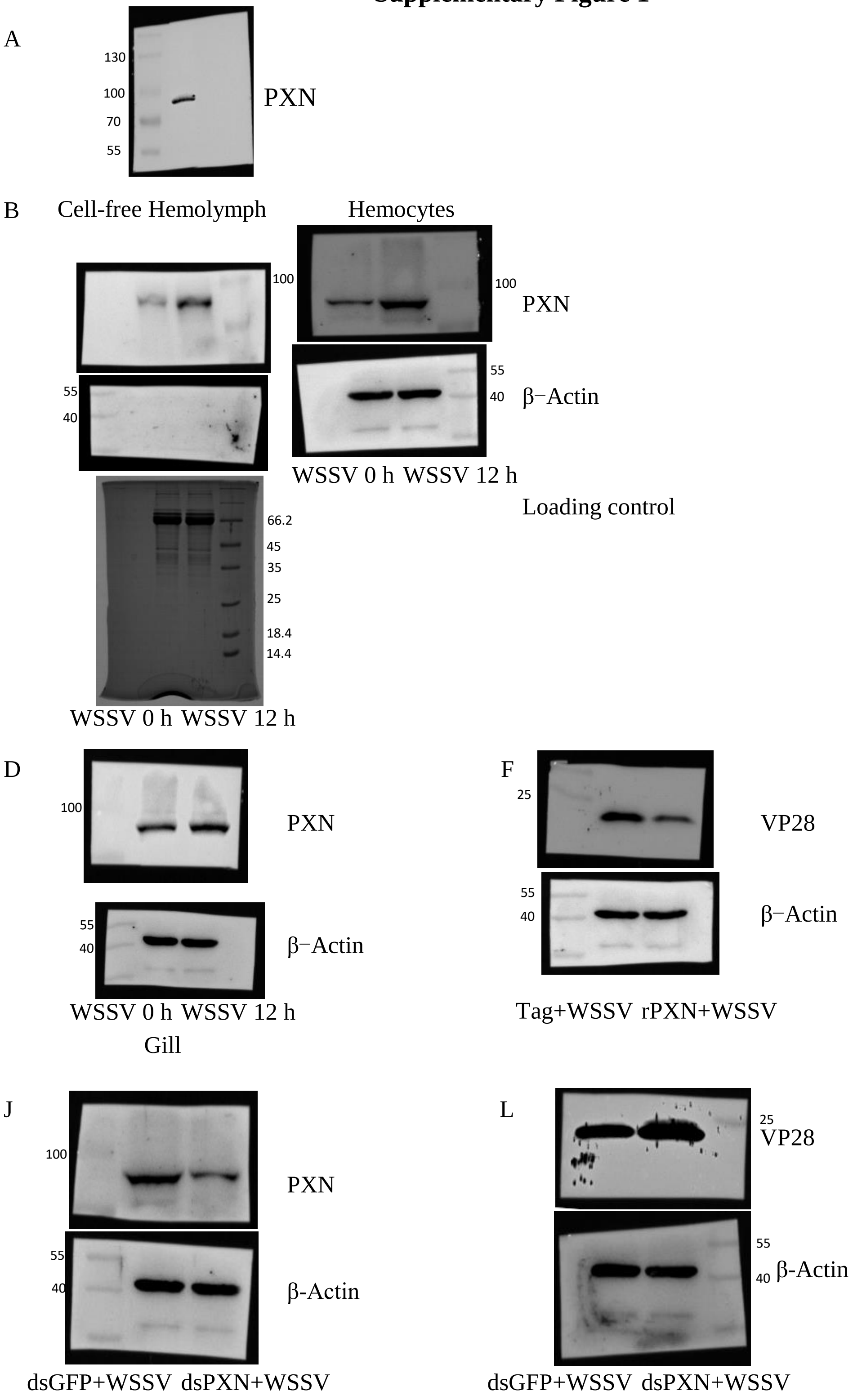

SFigure 1. The antiviral functions of PXN in crayfish. (A) PXN in hemocytes was detected by Western blotting using anti-PXN serum as the primary antibody. (B) Protein expression levels of PXN in cell-free hemolymph and hemocytes after WSSV infection. The total protein loading of the cell-free hemolymph was analyzed via SDS-PAGE. Gel staining with Coomassie Brilliant Blue was used as the loading control to verify protein loading amounts for cell-free hemolymph samples while β-Actin served as the internal control for hemocytes samples. (D) The protein expression level of PXN in gills after WSSV infection. β-Actin served as internal control. (F) The protein expression level of VP28 in rPXN-treated crayfish after WSSV infection.

β-Actin served as internal control. (J) The protein expression level of PXN in PXN-RNAi crayfish after WSSV infection. β-Actin served as internal control. (L) The protein expression level of VP28 in PXN-RNAi crayfish after WSSV infection. β-Actin served as internal control. For the detection of different proteins (e.g., target protein and internal reference protein), the same protein sample was loaded onto separate gels. After electrophoresis and transfer, different independent blots were obtained.

Supplementary Figure 2

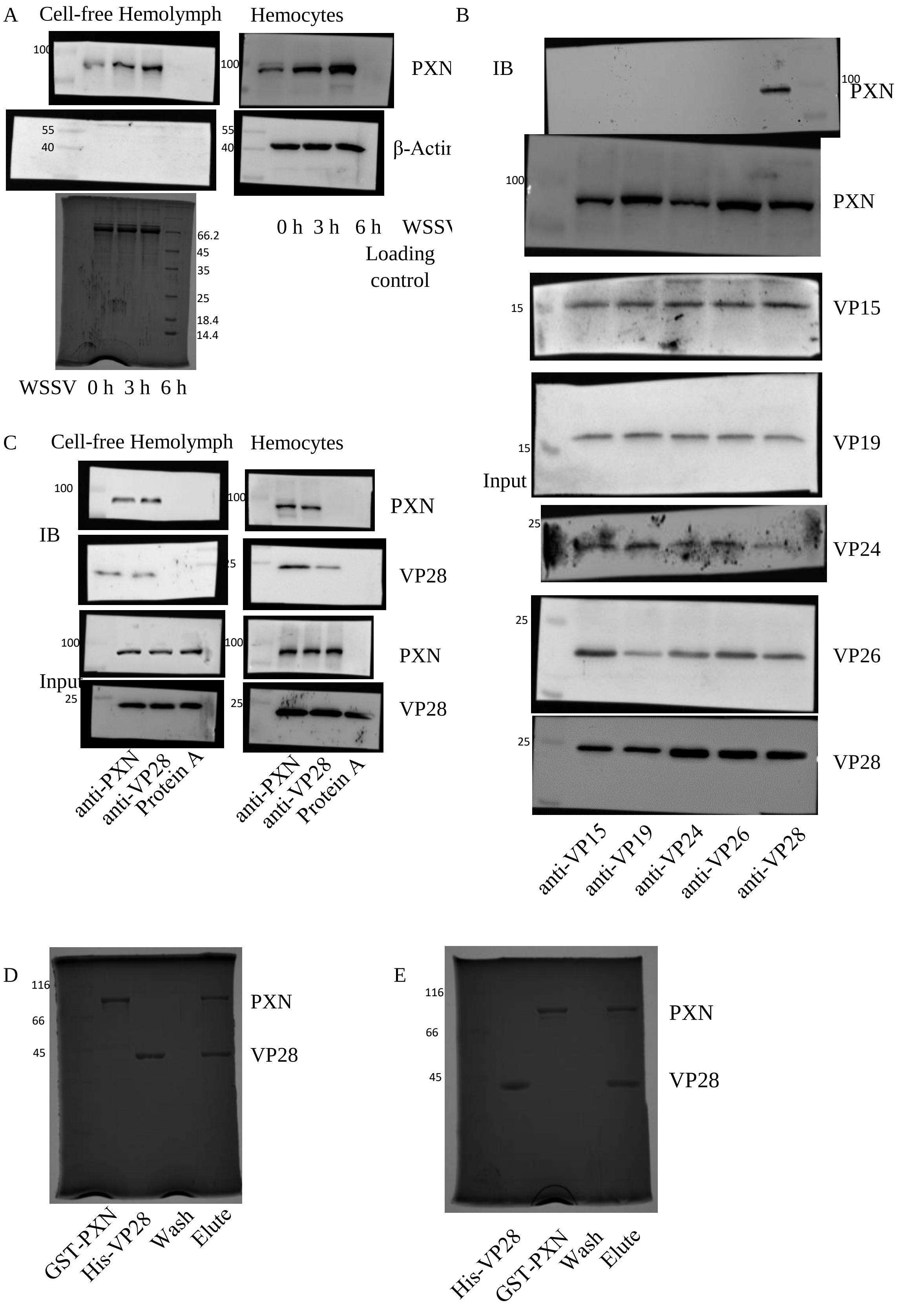

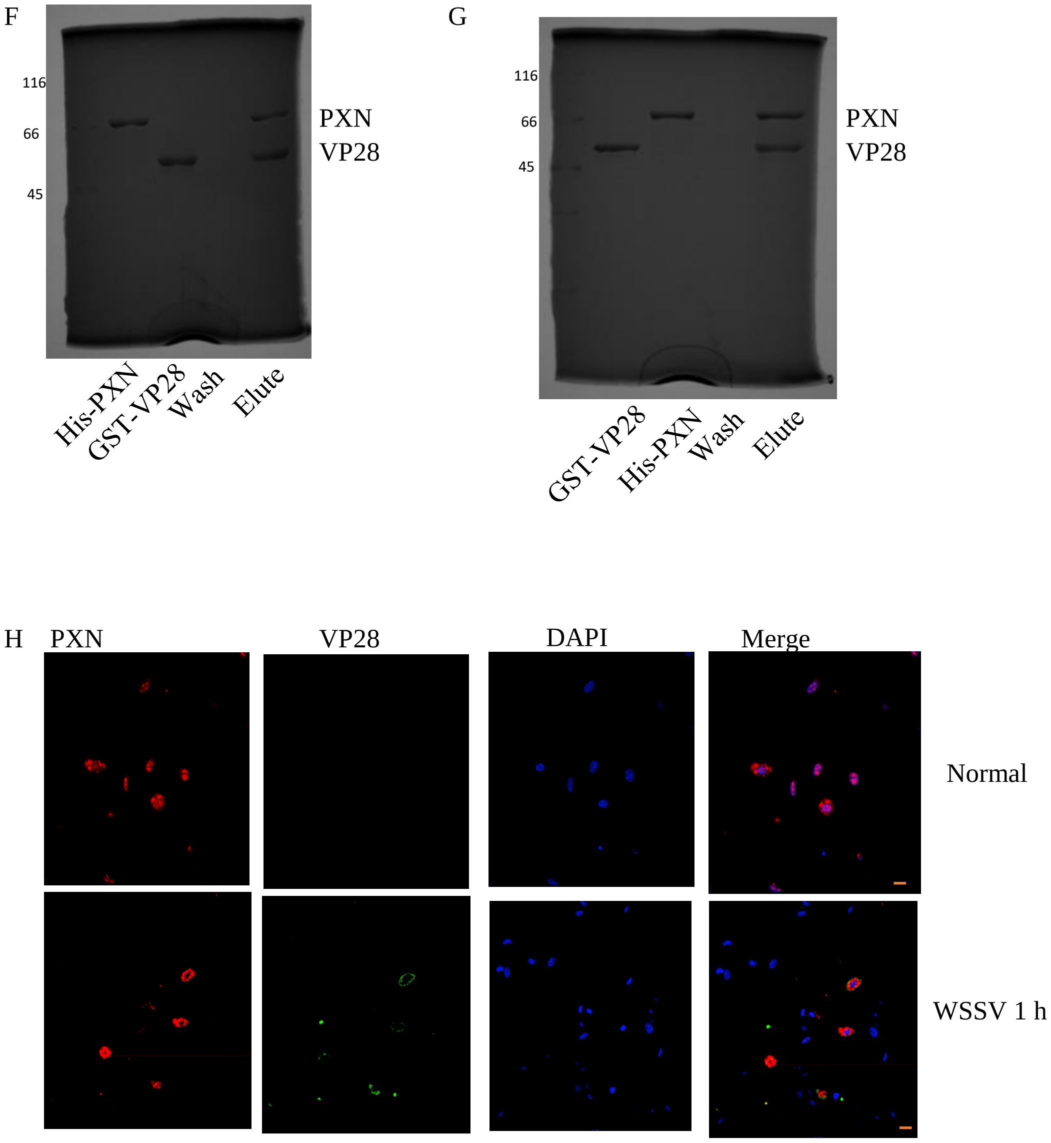

SFigure 2. Interaction between PXN and the envelope protein VP28 of WSSV. (A) Protein expression levels of PXN in cell-free hemolymph and hemocytes after WSSV infection. The total protein loading of the cell-free hemolymph was analyzed via SDS-PAGE. Gel staining with Coomassie Brilliant Blue was used as the loading control to verify protein loading amounts for cell-free hemolymph samples while β-Actin served as the internal control for hemocytes samples. (B) Interaction between WSSV major proteins (VP15, VP19, VP24, VP26 and VP28) and PXN. (C) Interaction between VP28 and PXN in cell-free hemolymph and hemocytes. (D-G) Interaction between rPXN and rVP28. (H) Colocalization of PXN and VP28 in hemocytes after WSSV infection. Scale bar=5 μm. For the detection of different proteins (e.g., target protein and internal reference protein), the same protein sample was loaded onto separate gels. After electrophoresis and transfer, different independent blots were obtained.

Supplementary Figure 3

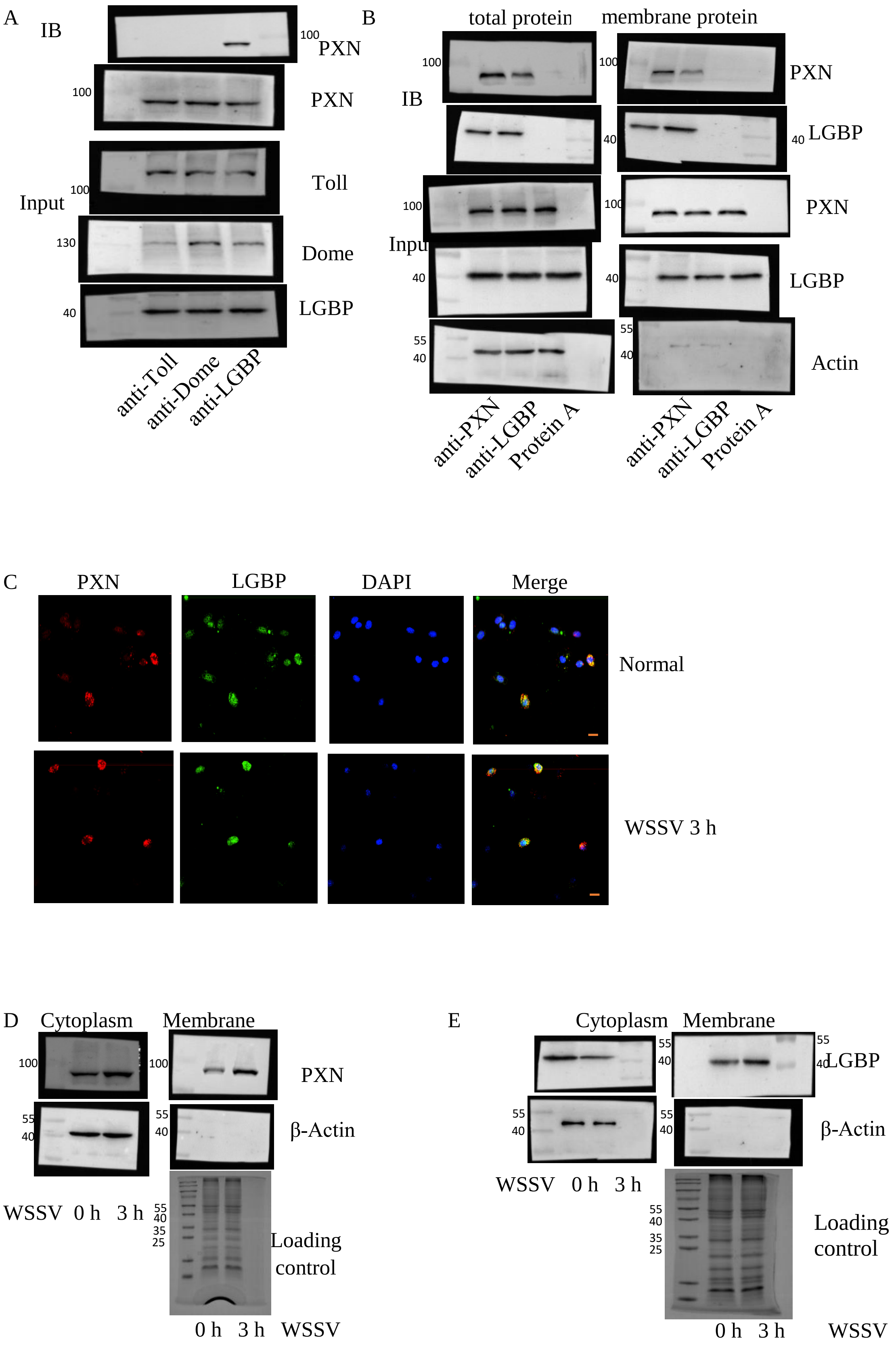

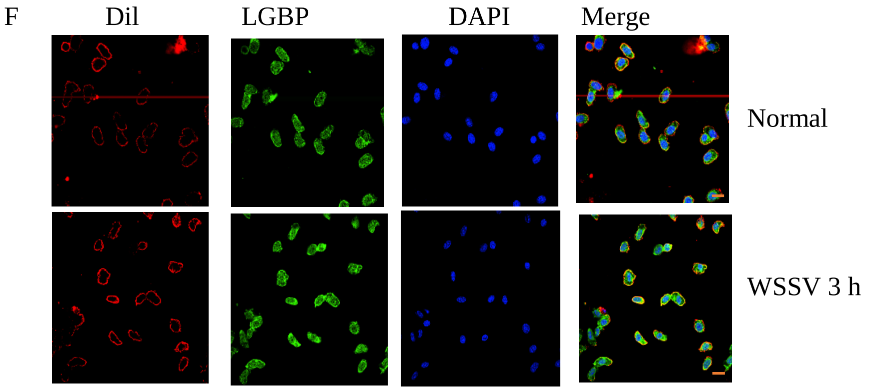

SFigure 3. Interaction between LGBP and PXN. (A) Interactions between Toll, Dome, LGBP and PXN. (B) Interaction between PXN and LGBP. (C) Colocalization of PXN and LGBP in hemocytes. (D) Subcellular localization of PXN in the cytoplasm and membrane after WSSV infection. Gel staining with Coomassie Brilliant Blue was used as the loading control to verify protein loading amounts for membrane samples while β-Actin served as the internal control for cytoplasm samples. (E) Subcellular localization of LGBP in the cytoplasm and membrane after WSSV infection. Gel staining with Coomassie Brilliant Blue was used as the loading control to verify protein loading amounts for membrane samples while β-Actin served as the internal control for cytoplasm samples. (F) Colocalization of LGBP and the membrane in hemocytes. Scale bar=5 μm. The total protein loading of the cell membrane was analyzed via SDS-PAGE. The gel was stained with Coomassie Brilliant Blue as a loading control. For the detection of different proteins (e.g., target protein and internal reference protein), the same protein sample was loaded onto separate gels. After electrophoresis and transfer, different independent blots were obtained.

Supplementary Figure 4

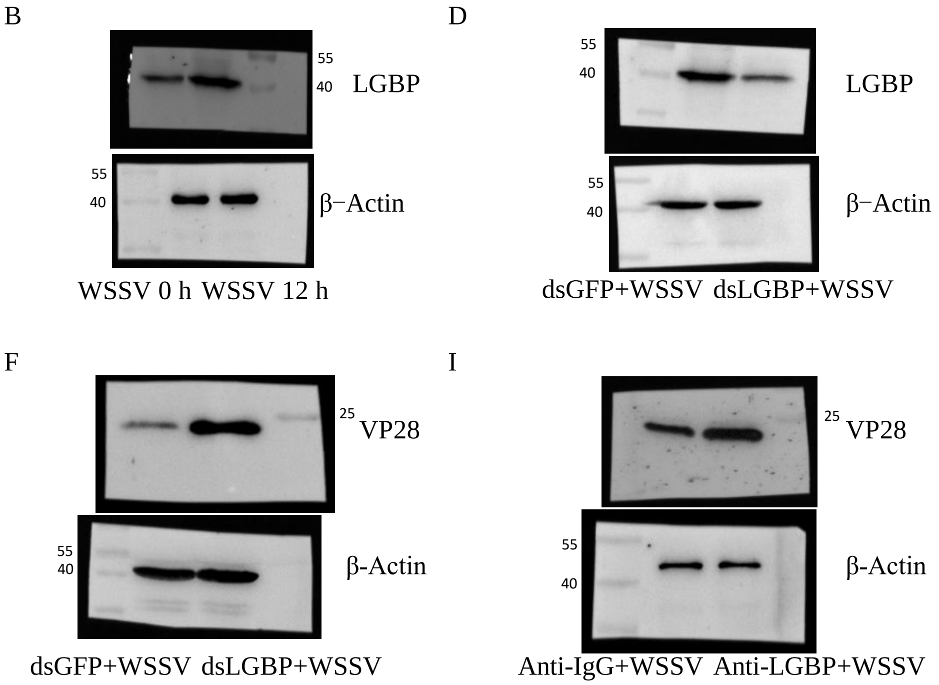

SFigure 4. The antiviral functions of LGBP. (B) Protein expression level of LGBP in hemocytes after WSSV infection. β-Actin served as internal control. (D) Protein expression level of LGBP in LGBP-RNAi-treated crayfish. β-Actin served as internal control. (F) The protein expression level of VP28 in LGBP-RNAi crayfish after WSSV infection. β-Actin served as internal control. (I) The protein expression level of VP28 in anti-LGBP-treated crayfish after WSSV infection. β-Actin served as internal control. For the detection of different proteins (e.g., target protein and internal reference protein), the same protein sample was loaded onto separate gels. After electrophoresis and transfer, different independent blots were obtained.

Supplementary Figure 5

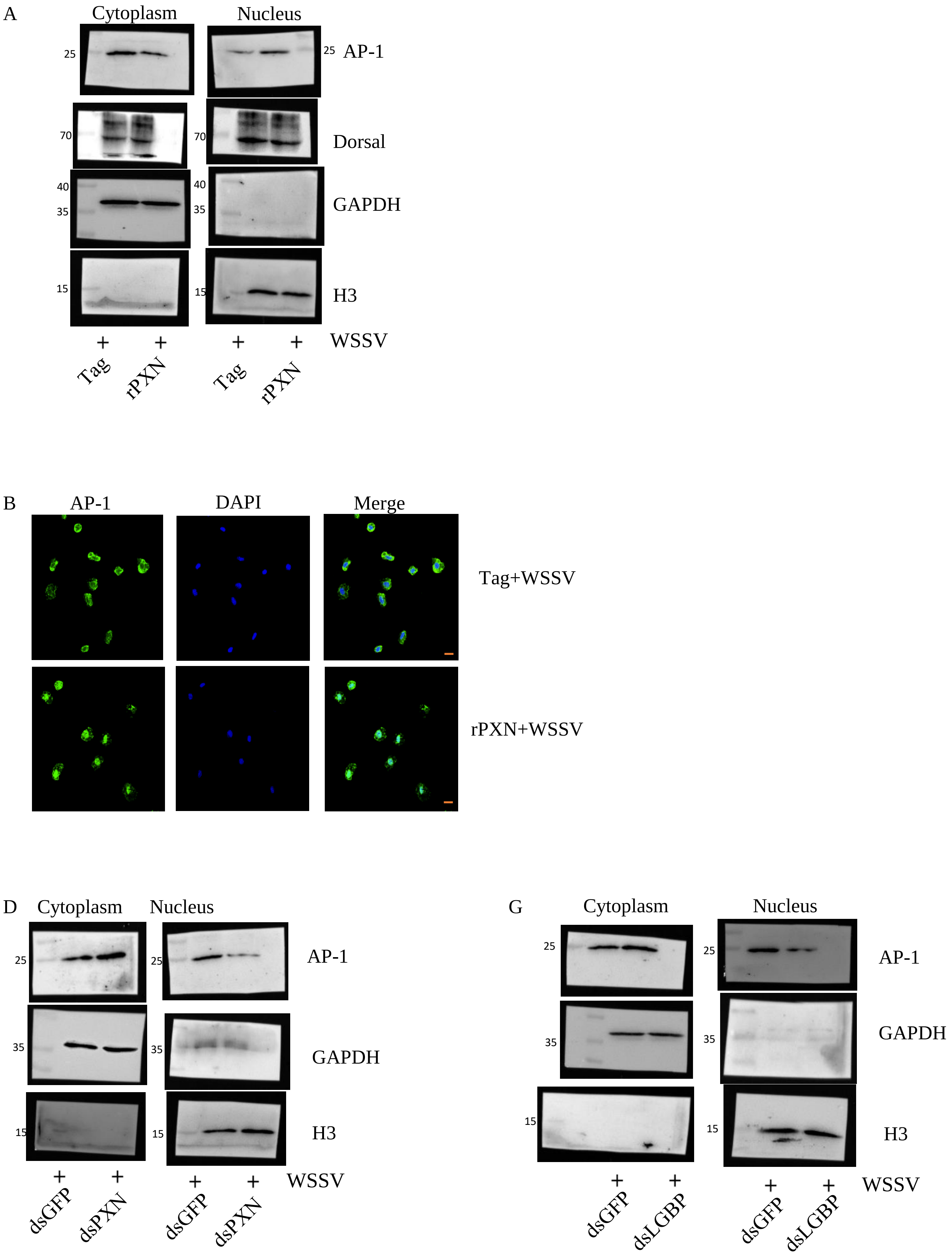

E

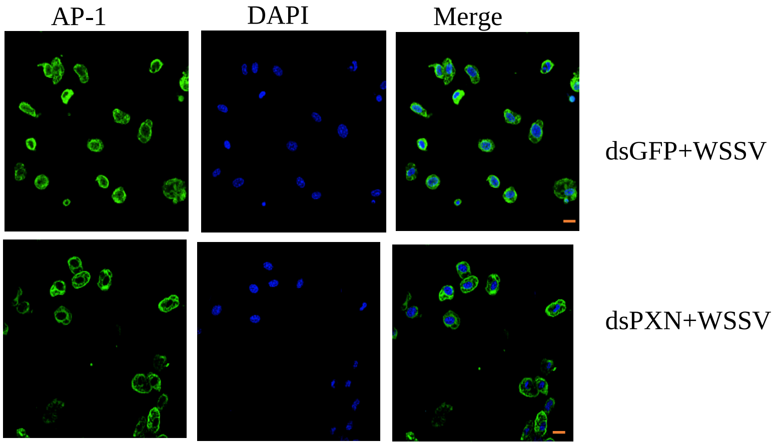
AP-1
DAPI
Merge
dsGFP+WSSV
dsPXN+WSSV

H

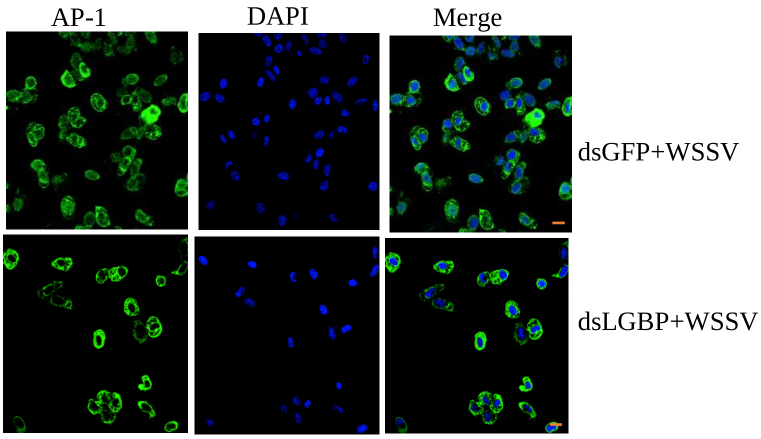
AP-1
DAPI
Merge
dsGFP+WSSV
dsLGBP+WSSV

J

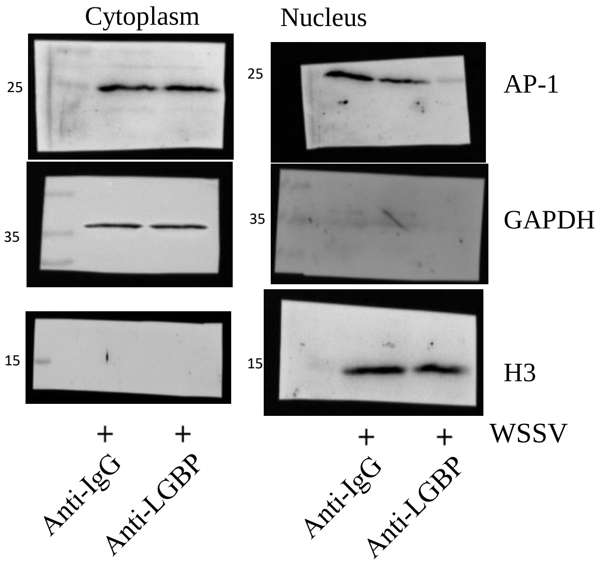
Cytoplasm
Nucleus
25
25
AP-1
35
35
GAPDH
15
15
H3
+
+
+
+
WSSV
Anti-IgG
Anti-LGBP
Anti-IgG
Anti-LGBP

K

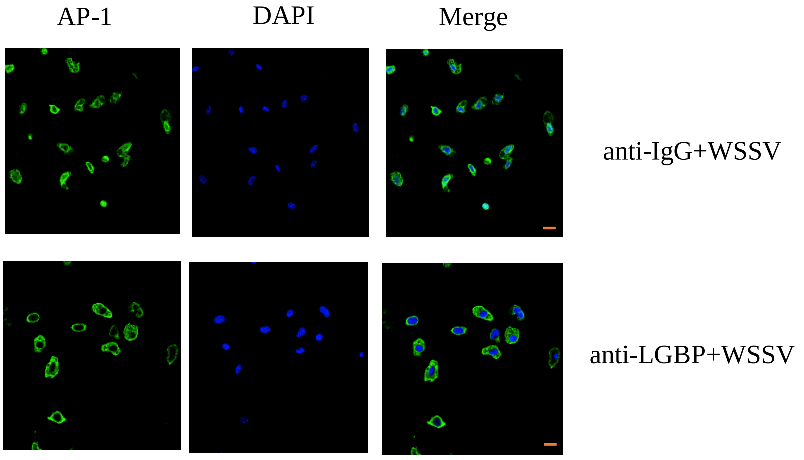

M

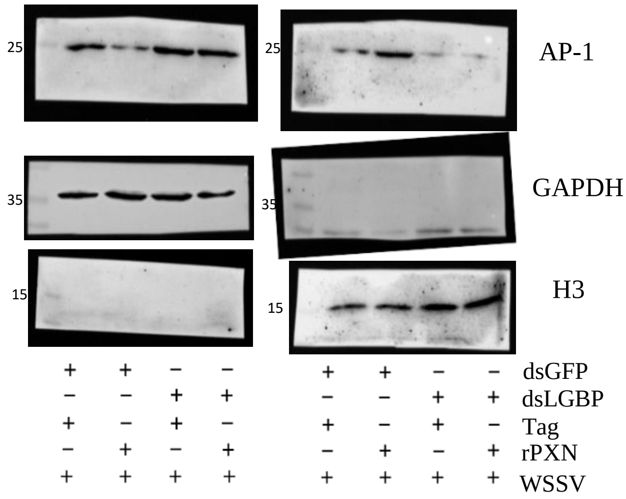

N

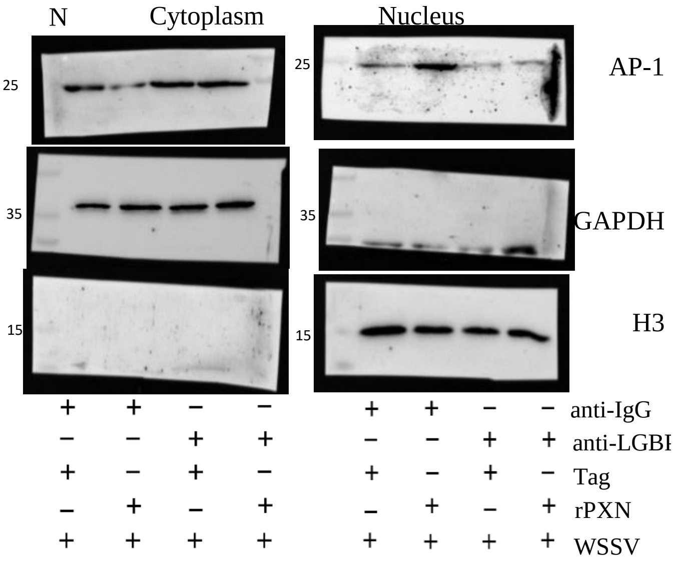

SFigure 5. Regulatory effects of PXN and LGBP on the nuclear translocation of AP-1 after WSSV infection. (A) Nuclear translocation of AP-1 and Dorsal in rPXN-treated crayfish after WSSV infection. (B) The nuclear translocation of AP-1 was detected by immunocytochemistry in rPXN-treated crayfish after WSSV infection. (D-E) Nuclear translocation of AP-1 in PXN-RNAi crayfish after WSSV infection. (G-H) Nuclear translocation of AP-1 in LGBP-RNAi crayfish after WSSV infection. (J-K) Nuclear translocation of AP-1 in anti-LGBP-treated crayfish after WSSV infection. (M-N) The subcellular distribution of AP-1 was analyzed in crayfish subjected to LGBP-RNAi or anti-LGBP treatment, followed by rPXN treatment and subsequent WSSV infection. H3 served as the internal control for nucleus samples while GAPDH served as the internal control for cytoplasm samples. For the detection of different proteins (e.g., target protein and internal reference protein), the same protein sample was loaded onto separate gels. After electrophoresis and transfer, different independent blots were obtained. Scale bar=5 μm.

Supplementary Figure 6

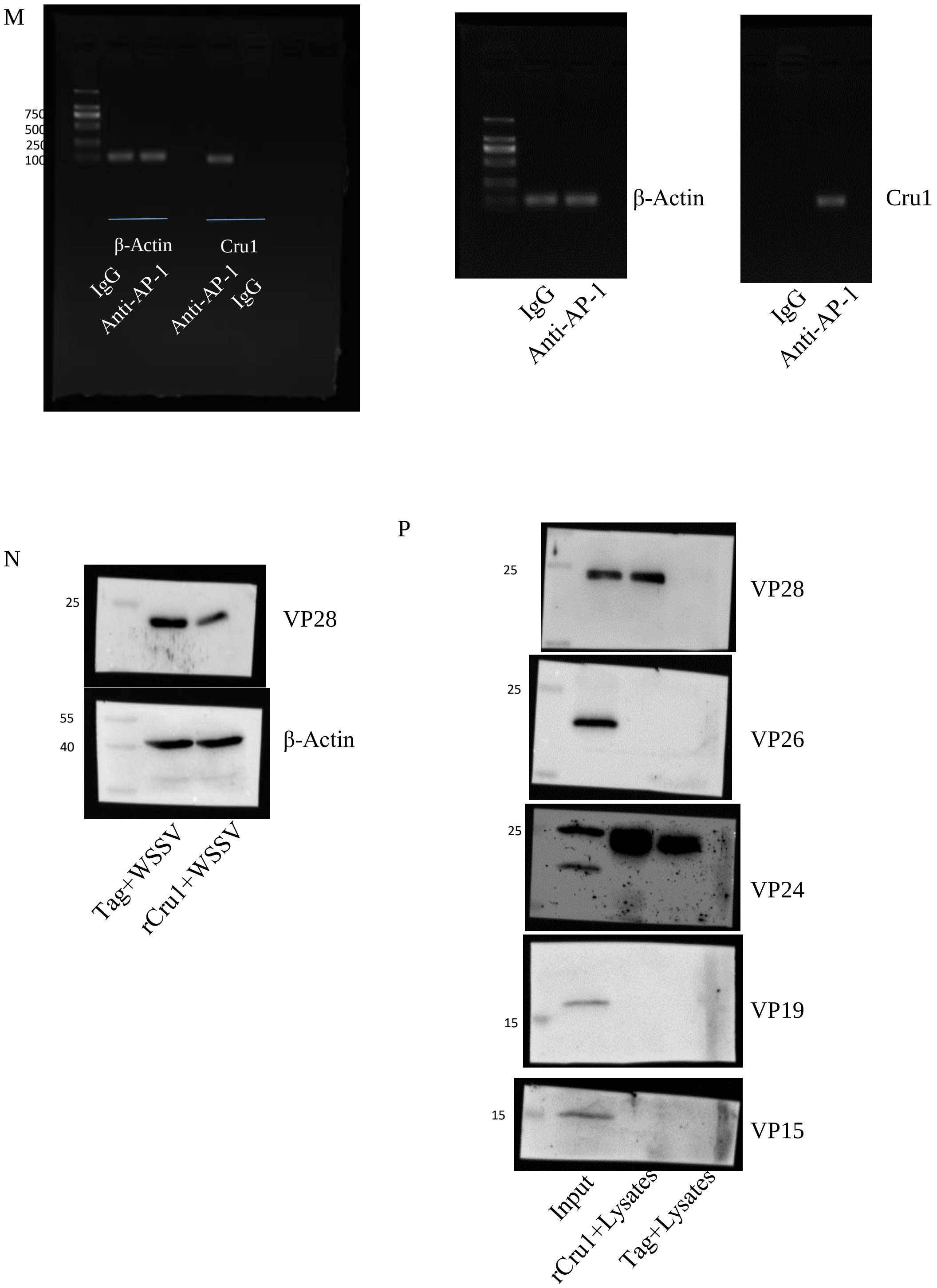

SFigure 6. Regulatory effects of PXN and LGBP on Cru1 expression and the antiviral functions of Cru1. (M) A ChIP assay was performed to assess the binding of AP-1 to the Cru1 promoter. β-Actin served as internal control. (N) The protein expression level of VP28 in rCru1-treated crayfish after WSSV infection. β-Actin served as internal control. (P) The interaction between rCru1 and native WSSV (VP15, VP19, VP24, VP26 and VP28) was detected. For the detection of different proteins (e.g., target protein and internal reference protein), the same protein sample was loaded onto separate gels. After electrophoresis and transfer, different independent blots were obtained.
