# Supplementary material for: The pxn-lgbp-ap-1 pathway restricts virus proliferation by inducing the expression of Cru1 in crayfish
Source: Commun Biol. 2025 Dec 3;8:1742. doi: 10.1038/s42003-025-09133-1 (PMC12675793; doi:10.1038/s42003-025-09133-1)
Supplement: Supplementary file 2 — Description of Additional Supplementary Files [file 42003_2025_9133_MOESM2_ESM.pdf]

Description of Additional Supplementary files

File name: Supplementary Data 1

Description: Numerical source data behind the graphs in this paper.

File name: Supplementary Information

Description: Supplementary Table 1, the uncropped blots, gels and immunofluorescence images. The uncropped blots, gels and immunofluorescence images in Supplementary Figure 1-6 correspond one-to-one with the blots, gels and immunofluorescence images in Figure 1-6 of the paper.
