# Supplementary material for: The pxn-lgbp-ap-1 pathway restricts virus proliferation by inducing the expression of Cru1 in crayfish
Source: Commun Biol. 2025 Dec 3;8:1742. doi: 10.1038/s42003-025-09133-1 (PMC12675793; doi:10.1038/s42003-025-09133-1)
Supplement: Supplementary file 4 — Reporting summary [file 42003_2025_9133_MOESM4_ESM.pdf]

nature portfolio

Corresponding author(s): DAPR COMMSBIO-25-6820C

Last updated by author(s): Sep 23, 2025

Reporting Summary

Nature Portfolio wishes to improve the reproducibility of the work that we publish. This form provides structure for consistency and transparency in reporting. For further information on Nature Portfolio policies, see our Editorial Policies and the Editorial Policy Checklist.

Statistics

For all statistical analyses, confirm that the following items are present in the figure legend, table legend, main text, or Methods section.

n/a	Confirmed	
☐	☒	The exact sample size (*n*) for each experimental group/condition, given as a discrete number and unit of measurement
☒	☐	A statement on whether measurements were taken from distinct samples or whether the same sample was measured repeatedly
☐	☒	The statistical test(s) used AND whether they are one- or two-sided<br>*Only common tests should be described solely by name; describe more complex techniques in the Methods section.*
☐	☒	A description of all covariates tested
☒	☐	A description of any assumptions or corrections, such as tests of normality and adjustment for multiple comparisons
☐	☒	A full description of the statistical parameters including central tendency (e.g. means) or other basic estimates (e.g. regression coefficient) AND variation (e.g. standard deviation) or associated estimates of uncertainty (e.g. confidence intervals)
☒	☐	For null hypothesis testing, the test statistic (e.g. *F*, *t*, *r*) with confidence intervals, effect sizes, degrees of freedom and *P* value noted<br>*Give P values as exact values whenever suitable.*
☒	☐	For Bayesian analysis, information on the choice of priors and Markov chain Monte Carlo settings
☒	☐	For hierarchical and complex designs, identification of the appropriate level for tests and full reporting of outcomes
☒	☐	Estimates of effect sizes (e.g. Cohen's *d*, Pearson's *r*), indicating how they were calculated

Our web collection on statistics for biologists contains articles on many of the points above.

Software and code

Policy information about availability of computer code

Data collection	The nucleotide sequence of PXN and LGBP from species were obtained from the National Center for Biotechnology Information (https://www.ncbi.nlm.nih.gov/). The RT-PCR data were collected using GenoSens Capture Software. The Western blot data were collected using SHST Capture Software. Fluorescence was observed under the Andor Dragonfly High Speed Confocal Microscope (Dragonfly). The Immunocytochemistry data were collected using Fusion Software. The qRT-PCR data were collected using QuantStudio Design & Analysis Software.
Data analysis	The domain architecture was predicted using SMART (http://smart.embl-heidelberg.de/). The promoter sequence was analyzed using Promoter Scan online (https://www-bimas.cit.nih.gov/molbio/proscan/). Statistical analyses were performed using GraphPad Prism 8.0 software. The gray value of the band was analyzed using the Wright Cell Imaging Facility ImageJ software. Group comparisons analyzed by Student's t-test.

For manuscripts utilizing custom algorithms or software that are central to the research but not yet described in published literature, software must be made available to editors and reviewers. We strongly encourage code deposition in a community repository (e.g. GitHub). See the Nature Portfolio guidelines for submitting code & software for further information.

Data

Policy information about availability of data

All manuscripts must include a data availability statement. This statement should provide the following information, where applicable:

- Accession codes, unique identifiers, or web links for publicly available datasets
- A description of any restrictions on data availability
- For clinical datasets or third party data, please ensure that the statement adheres to our policy

All raw data are available in Supplemental Data.

Research involving human participants, their data, or biological material

Policy information about studies with human participants or human data. See also policy information about sex, gender (identity/presentation), and sexual orientation and race, ethnicity and racism.

Reporting on sex and gender	not applicable
Reporting on race, ethnicity, or other socially relevant groupings	not applicable
Population characteristics	not applicable
Recruitment	not applicable
Ethics oversight	not applicable

Note that full information on the approval of the study protocol must also be provided in the manuscript.

Field-specific reporting

Please select the one below that is the best fit for your research. If you are not sure, read the appropriate sections before making your selection.

☒ Life sciences ☐ Behavioural & social sciences ☐ Ecological, evolutionary & environmental sciences

For a reference copy of the document with all sections, see nature.com/documents/nr-reporting-summary-flat.pdf

Life sciences study design

All studies must disclose on these points even when the disclosure is negative.

Sample size	The crayfish P. clarkii (10-15 g each) were used in this study were all healthy..
Data exclusions	No date were excluded from the analyses.
Replication	All attempts at replication were successful.
Randomization	The crayfish (similar in color, size and health condition) were randomly divided into the experimental group and the control group.
Blinding	The group allocation during data collection and analysis were blinded.

Reporting for specific materials, systems and methods

We require information from authors about some types of materials, experimental systems and methods used in many studies. Here, indicate whether each material, system or method listed is relevant to your study. If you are not sure if a list item applies to your research, read the appropriate section before selecting a response.

Materials & experimental systems

n/a	Involved in the study	
☐	☒	Antibodies
☒	☐	Eukaryotic cell lines
☒	☐	Palaeontology and archaeology
☐	☒	Animals and other organisms
☒	☐	Clinical data
☒	☐	Dual use research of concern
☒	☐	Plants

Methods

n/a	Involved in the study	
☒	☐	ChIP-seq
☒	☐	Flow cytometry
☒	☐	MRI-based neuroimaging

Antibodies

Antibodies used	goat anti-rabbit Alexa Fluor 488 (Abbkine， A23220); HRP-conjugated goat anti-rabbit IgG (Proteintech， SA00001-2); The primary antibodies used in this article are all polyclonal antibodies previously prepared by our laboratory (as published in previous articles).
Validation	The antibodies prepared and verified in this study have been proven to be usable in published papers (The HMGBa-HSP70-ATF4-β Axis Restricts White Spot Syndrome Virus Infection in Crayfish. J Immunol. 2024; PcTrim prevents early infection with white spot syndrome virus by inhibiting AP1-induced endocytosis. Cell communication and signaling. 2023). Other antibodies are described on the supplier's website.

Animals and other research organisms

Policy information about studies involving animals; ARRIVE guidelines recommended for reporting animal research, and Sex and Gender in Research

Laboratory animals	The experimental animals involved in this study include Procambarus clarkii and rabbit.
Wild animals	This study did not involve wild animals.
Reporting on sex	Sex was not considered in this study.
Field-collected samples	This study did not involve samples collected from the field.
Ethics oversight	The study was approved by the Committee on the Animal Ethics of Shandong Agricultural University. Experiments were carried out in accordance with the approved guidelines (No. SDAUA-2024-065).

Note that full information on the approval of the study protocol must also be provided in the manuscript.

Plants

Seed stocks	not applicable
Novel plant genotypes	not applicable
Authentication	not applicable
